# New insight into the genetic basis of oil content based on noninvasive three-dimensional phenotyping and tissue-specific transcriptome in *Brassica napus*

**DOI:** 10.1186/s13068-023-02324-0

**Published:** 2023-05-23

**Authors:** Liangxing Guo, Hongbo Chao, Yongtai Yin, Huaixin Li, Hao Wang, Weiguo Zhao, Dalin Hou, Libin Zhang, Chunyu Zhang, Maoteng Li

**Affiliations:** 1 Department of Biotechnology, College of Life Science and Technology, Huazhong University of Science and Technology, Wuhan, 430074 China; 2 Hybrid Rapeseed Research Center of Shaanxi Province, Shaanxi Rapeseed Branch of National Centre for Oil Crops Genetic Improvement, Yangling, 712100 China; 3 National Key Lab of Crop Genetic Improvement and College of Plant Science and Technology, Huazhong Agricultural University, Wuhan, 430070 China

**Keywords:** *Brassica napus*, Lipid distribution, Oil content, Three-dimensional (3D) phenotyping, Tissue-specific QTLs, Magnetic resonance imaging (MRI), Tissue-specific transcriptome

## Abstract

**Background:**

Increasing seed oil content is the most important breeding goal in *Brassica napus*, and phenotyping is crucial to dissect its genetic basis in crops. To date, QTL mapping for oil content has been based on whole seeds, and the lipid distribution is far from uniform in different tissues of seeds in *B. napus*. In this case, the phenotype based on whole seeds was unable to sufficiently reveal the complex genetic characteristics of seed oil content.

**Results:**

Here, the three-dimensional (3D) distribution of lipid was determined for *B. napus* seeds by magnetic resonance imaging (MRI) and 3D quantitative analysis, and ten novel oil content-related traits were obtained by subdividing the seeds. Based on a high-density genetic linkage map, 35 QTLs were identified for 4 tissues, the outer cotyledon (OC), inner cotyledon (IC), radicle (R) and seed coat (SC), which explained up to 13.76% of the phenotypic variation. Notably, 14 tissue-specific QTLs were reported for the first time, 7 of which were novel. Moreover, haplotype analysis showed that the favorable alleles for different seed tissues exhibited cumulative effects on oil content. Furthermore, tissue-specific transcriptomes revealed that more active energy and pyruvate metabolism influenced carbon flow in the IC, OC and R than in the SC at the early and middle seed development stages, thus affecting the distribution difference in oil content. Combining tissue-specific QTL mapping and transcriptomics, 86 important candidate genes associated with lipid metabolism were identified that underlie 19 unique QTLs, including the fatty acid synthesis rate-limiting enzyme-related gene *CAC2*, in the QTLs for OC and IC.

**Conclusions:**

The present study provides further insight into the genetic basis of seed oil content at the tissue-specific level.

**Supplementary Information:**

The online version contains supplementary material available at 10.1186/s13068-023-02324-0.

## Highlight

Three-dimensional phenotyping, QTL identification with a high-density genetic linkage map and transcriptomic analysis were combined to dissect the genetic architecture of oil content in different seed tissues of *Brassica napus*.

**Supplementary Information:**

The online version contains supplementary material available at 10.1186/s13068-023-02324-0.

## Background

Rapeseed (*Brassica napus*) is one of the most important oilseed crops in the world. Increasing the oil content is the most important goal for rapeseed breeding at present [1, 2]. Comprehensively understanding the oil content and lipid distribution in seeds of *B. napus* is important for breeding. Although some genes that encode lipid biosynthesis in plants have been identified [3–8], the genetic basis and molecular regulation of lipid variation in different seed tissues in *B. napus* are poorly understood.

The analytical determination of oil content in rapeseed has been traditionally accomplished by the Soxhlet-based extraction method and residual method with organic solvents as the extraction medium [9], and supercritical fluid extraction was performed with carbon dioxide as the extraction solvent [10]. The seed lipid could also be analyzed using gas chromatography [11]. The abovementioned oil content measurement methods are all carried out after oil dissection or extraction [9–11]. However, the destruction of seeds in these methods is inappropriate if these materials need to be cultivated for other studies after oil content measurement [12]. Thus, some rapid, simple, and nondestructive methods, such as pulsed nuclear magnetic resonance [13] and near-infrared spectroscopy [14] have been developed for the determination of oil content and have been widely used for the evaluation of the oil content in seeds [12, 15–18]. However, the measurement and visualization of oil for specific seed tissues cannot be accomplished based on the two above methods. The conventional procedures used for lipid visualization, such as Nile red and Sudan black B [19], are based on dyes in two dimensions and are destructive [19]. Mass spectrometry imaging (MSI) has been used to visualize the lipid composition in both plant and animal material [20–22]. However, quantification in MSI is still a major hurdle, and the methods are destructive [23]. Recently, an approach that allows the rapid, noninvasive and quantitative visualization of lipids in living seeds using frequency-selected MRI has been reported [24, 25], and this method could provide quantitative lipid maps with a resolution close to the cellular level [26]. Based on MRI, each radiofrequency (RF) pulse excites a narrow slice in the two-dimensional (2D) sequence, whereas each RF pulse excites the entire imaging volume, and encoding is used to discriminate spatially in a three-dimensional (3D) sequence, which is helpful for fully revealing the lipid distribution in seeds and providing more detailed 3D lipid quantitative information.

Oil content is a typical quantitative trait that is controlled by a large number of genes with mainly additive and few epistatic gene actions [27–30]. QTL mapping is an effective approach to dissect the genetic mechanisms of such complex quantitative traits [31], and numerous QTLs for oil content have been identified in *B. napus* [27, 28, 32–42]. Lipid biosynthesis is complex and regulated by various biosynthetic enzymes and transcription factors [43], which vary dramatically from tissue to tissue within seeds [44]. Therefore, dissecting the genetic basis for oil content differences in different tissues of seeds is of great significance for further increasing the oil content in rapeseed. Recently, Lu et al. [45] revealed the complex spatial distribution patterns of lipids and transcripts by integrating the results from matrix-assisted laser desorption/ionization-mass spectrometry imaging (MALDI-MSI) of lipids in situ, lipidome profiling of extracts from seed tissues, and tissue-specific transcriptome analysis [45]. However, little is known about the spatial distribution patterns and genetic mechanisms of lipids in different seed tissues in *B. napus* at the population level.

Phenotyping is crucial to reveal genetic variation accompanied by genomics in crops. With the development of genome sequencing technology, precise assessment of plant phenotypes is falling behind the ability to characterize genotypes [46–48]. To date, the phenotypic data of oil content for QTL mapping in rapeseed have all been based on whole seeds [28, 39–42, 49]. As a result, few new loci have been identified and utilized. If the oil content of different tissues is separated as an independent phenotype for QTL mapping, it would reveal the genetic mechanism of lipid accumulation differences in different tissues of seeds and provide new ideas for breeding to improve oil content. Recently, linkage mapping combined with high-throughput sequencing has been applied to dissect genetic mechanisms and is considered to be an effective way to identify candidate genes. For example, 24 genes were identified by combining SNP-trait association analysis and transcriptome sequencing for resistance to Sclerotinia stem rot in *B. napus* [50]. DEGs were integrated with association mapping and linkage analysis to confirm their roles in the growth process, and 12 candidate genes associated with growth period traits were found [51, 52]. *BnaC07. CCR-LIKE* (*CCRL*) and *BnaTT8s* play key roles in the determination of seed coat content by modulating lignin biosynthesis by combining transcriptome-wide association studies and correlation networks of seed coat content-related gene modules.

The present study visualized the 3D distribution of lipids and obtained the actual lipid content in different tissues of seeds in a double haploid mapping population of *B. napus* using the noninvasive MRI technique in a 30 μm × 30 μm × 30 μm voxel with a 3D-FISP sequence. A total of 10 traits related to oil content in four subdivided tissues (seed coat (SC), outer cotyledon (OC), inner cotyledon (IC), and radicle (R)) of seeds were obtained. Then, QTL mapping for oil content-related traits was implemented for the four subdivided tissues of seeds. Furthermore, tissue-specific transcriptome sequencing from two developmental stages (24 and 33 days after flowering (DAF)) of two parents with high oil content differences was employed to detect the gene expression characteristics of IC, OC, R and SC and assist in candidate gene identification. Finally, by combining QTL mapping, bulked segregant analysis (BSA) and tissue-specific transcriptomic analysis, common and specific candidates involved in lipid biosynthesis in different seed tissues were identified. This study provides new insights for further improving the oil content of seeds in *B. napus*.

## Results

### Using MRI and 3D restructuring to dissect the tissue-specific distribution of lipid in *B. napus* seeds

**Fig. 1:**
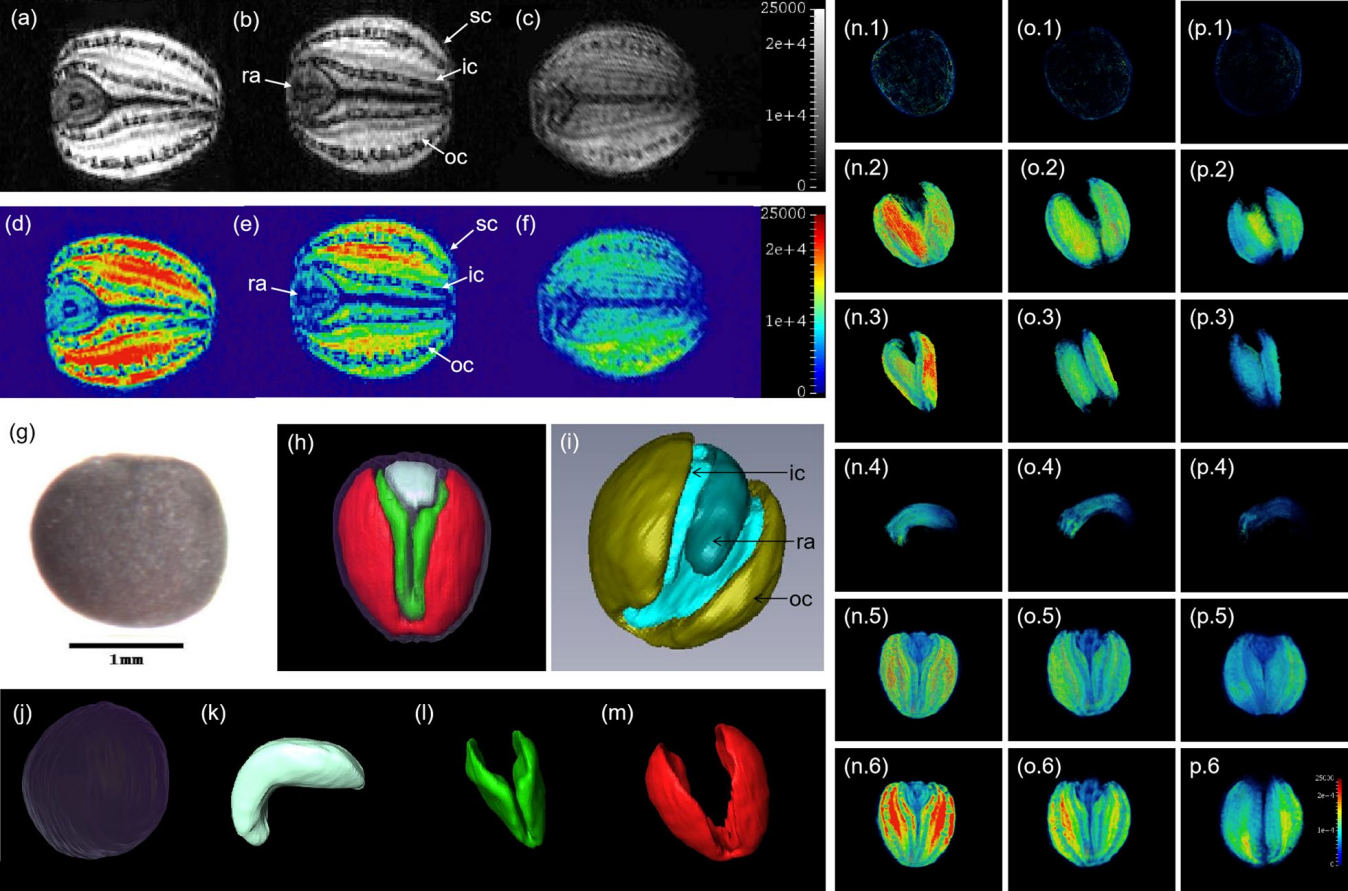
Quantitative imaging of lipids in rapeseed seeds with different oil content. **a**, **b** and **c** represent the final lipid distribution shown as a gray value (signal intensity), and **d**, **e** and **f** represent the final lipid distribution of seeds shown as a color coded with high oil content seeds, middle oil content seeds and low oil content seeds, respectively. **g**, **h** and **i** represent the original rapeseed seed and the reconstruction of the rapeseed seed. **j**, **k**, **l** and **m** represent the reconstruction of the seed coat, radicle, inner cotyledon and outer cotyledon. **n**, **o** and **p** represent the distribution of lipids in different tissues of seeds with high, middle and low oil content; 1, 2, 3, 4, 5 and 6 represent seed coat, outer cotyledon, inner cotyledon, radicle, whole seed and seed section, respectively. *sc* seed coat, *oc* outer cotyledon, *ic* inner cotyledon, *r* radicle

Mature *B. napus* seeds with high (51.47%), middle (46.90%) and low (40.10%) oil content were selected to analyze the lipid distribution using MRI. Images of lipids with a voxel resolution of 30 µm × 30 µm × 30 µm were obtained (Fig. 1a–c and d–f and n–p), and the SC, OC, IC and R tissues could be distinguished clearly (Fig. 1h, i). The results showed that the lipid signal intensity in the four subdivided tissues was significantly different, and a clear reduction in the direction from the OC to IC, R and SC was observed (Fig. 1a–c and d–f).

To better understand the distribution of lipid and calculate oil content in different tissues in seeds, 3D reconstruction was implemented combining MRI images and Amira 5.4.0 software. The whole seed (Fig. 1g, h) could be clearly separated into SC (Fig. 1j), OC (Fig. 1m), IC (Fig. 1l) and R (Fig. 1k) at the 3D level, and the volume of each tissue could be calculated. The total oil content (TO) and mean oil content (MO) of different tissues could be calculated based on the calibration curve (*r*^2^ = 0.99473) established based on the lipid signal intensity and lipid content measured by gas chromatography. The lipid signal intensity in different tissues was different (Fig. 1a–f and Fig. 1n–p), which indicated that there was different oil content in different types of seed tissues. These results indicated that through MRI and 3D reconstruction, lipid density and content in four fine tissues of *B. napus* seed could be quickly and accurately quantified without destroying the seed structure.

### Noninvasive 3D lipid phenotyping for four subdivided tissues in the KN segregating population

**Fig. 2:**
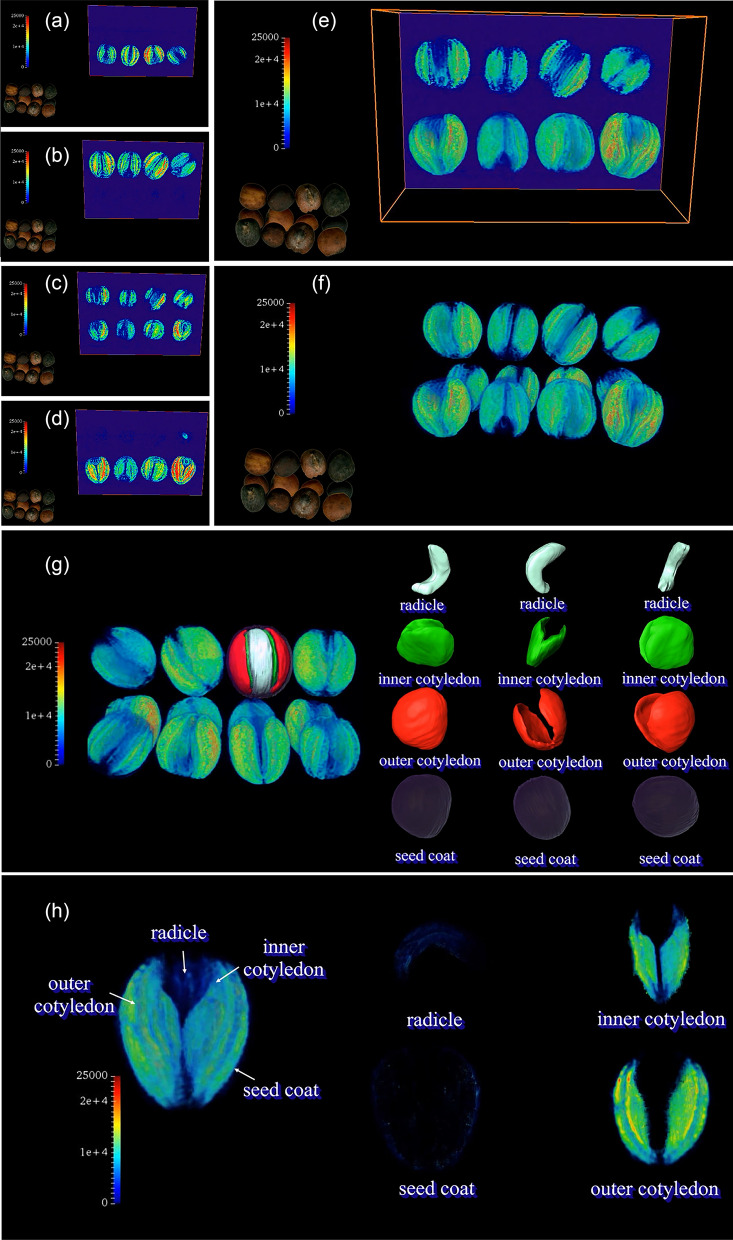
Quantitative imaging of lipids in rapeseed seeds of the KN DH population. **a**, **b**, **c**, and **d** represent the imaging process of lipids in seeds, and **e** and **f** represent the three-dimensional reconstruction of seeds based on lipid imaging. **g** Three-dimensional reconstruction of different seed tissues, including the seed coat, outer cotyledon, inner cotyledon, and radicle. **h** represents the whole seed and segmentation of different issues of seeds based on lipid imaging

**Fig. 3:**
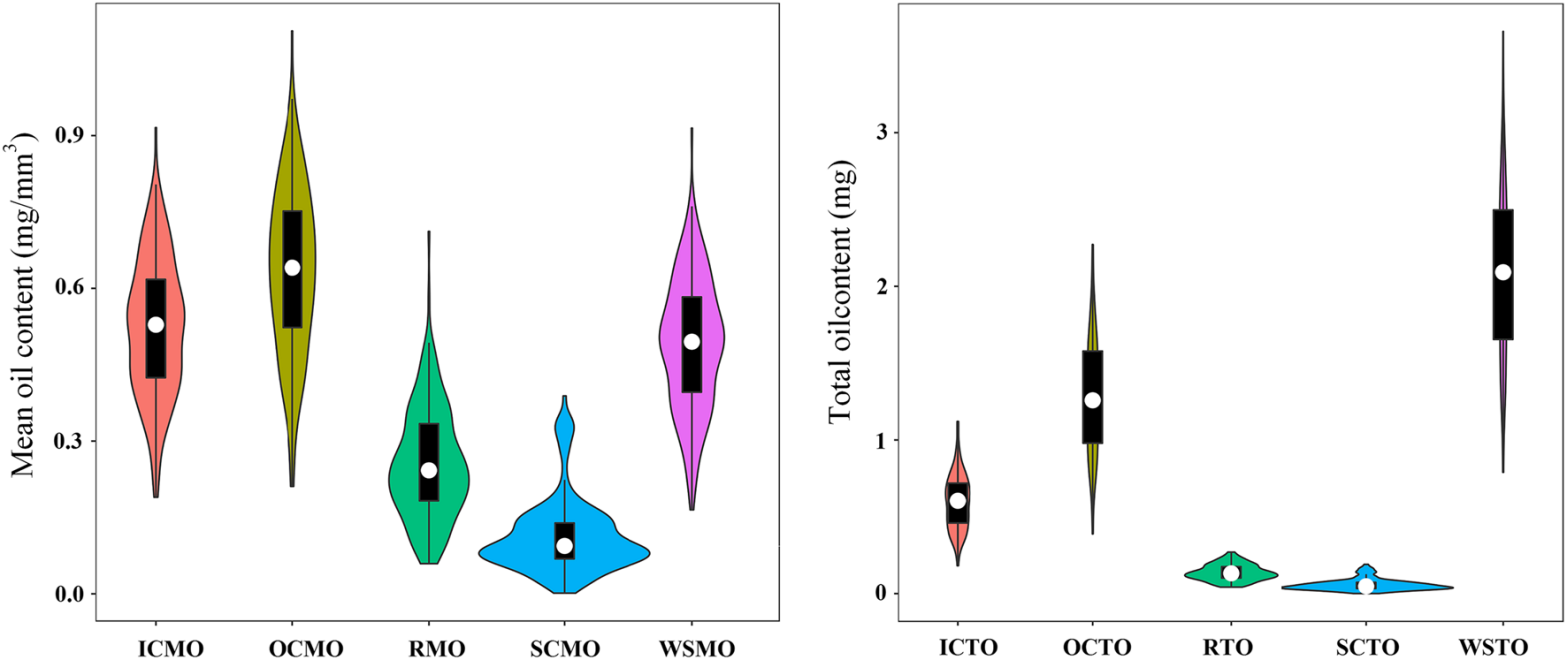
Frequency distribution of oil content in different tissues of seeds in the KN population. *SCMO* seed coat mean oil content, mg/mm^3^; *ICMO* inner cotyledon mean oil content, mg/mm^3^; *OCMO* outer cotyledon mean oil content, mg/mm^3^; *RMO* radicle mean oil content, mg/mm^3^; *SCTO* seed coat total oil content, mg; *ICTO* inner cotyledon total oil content, mg; *OCTO* outer cotyledon total oil content, mg; *RTO* radicle total oil content, mg; *WSTO* whole seed oil content, mg. The distribution density is overlaid

The abovementioned method was used for noninvasive phenotyping analysis for 200 DH lines in the KN population [49]. Finally, 10 novel traits related to oil content for different tissues of *B. napus* seeds, including OCTO, ICTO, RTO, SCTO, OCMO, ICMO, RMO, SCMO, WSTO and WSMO, were obtained. The detailed operating procedures of the MRI and Amira 5.4.0 are provided in the Methods section and supplementary video (Additional file 3: Video S1; Fig. 2). Based on the novel phenotypic data, a wide range of variation and transgressive segregation for 10 traits were observed in the KN population (Additional file 1: Fig. S2; Additional file 2: Table S1; Fig. 3). WSTO ranged from 0.79 mg to 3.66 mg with an average of 2.07 mg. For the four subdivided tissues, OC had the highest oil content (average of 1.28 mg, ranging from 0.39 to 2.27 mg), followed by IC (average of 0.60 mg, ranging from 0.18 to 1.12 mg), R (average of 0.14 mg, ranging from 0.04 to 0.27 mg) and SC (average of 0.058 mg, ranging from 0 to 0.19 mg). For the MO in the KN population, OC had the highest mean oil content, with an average of 0.63 mg/mm^3^ (ranging from 0.21 mg/mm^3^ to 1.11 mg/mm^3^), followed by IC (an average of 0.52 mg/mm^3^, ranging from 0.19 to 0.92 mg/mm^3^), R (an average of 0.254 mg/mm^3^, ranging from 0.06 to 0.71 mg/mm^3^) and SC (an average of 0.113 mg/mm^3^, ranging from 0 to 0.39 mg/mm^3^). The frequency distribution of 10 novel traits showed continuous variation in the KN population (Fig. 3), suggesting a polygenic effect of oil content in different seed tissues. The correlations among these 10 traits in the KN population were also calculated (Additional file 2: Table S2; Additional file 1: Fig. S3). Regardless of the TO or MO, the correlation between OC, IC and the whole seed was the strongest. In particular, OCTO and ICTO had high correlations of 0.962 and 0.832 with WSTO and WSMO, respectively.

### QTL mapping for oil content-related traits of subdivided tissues in *B. napus* seeds based on 3D phenotyping

**Fig. 4:**
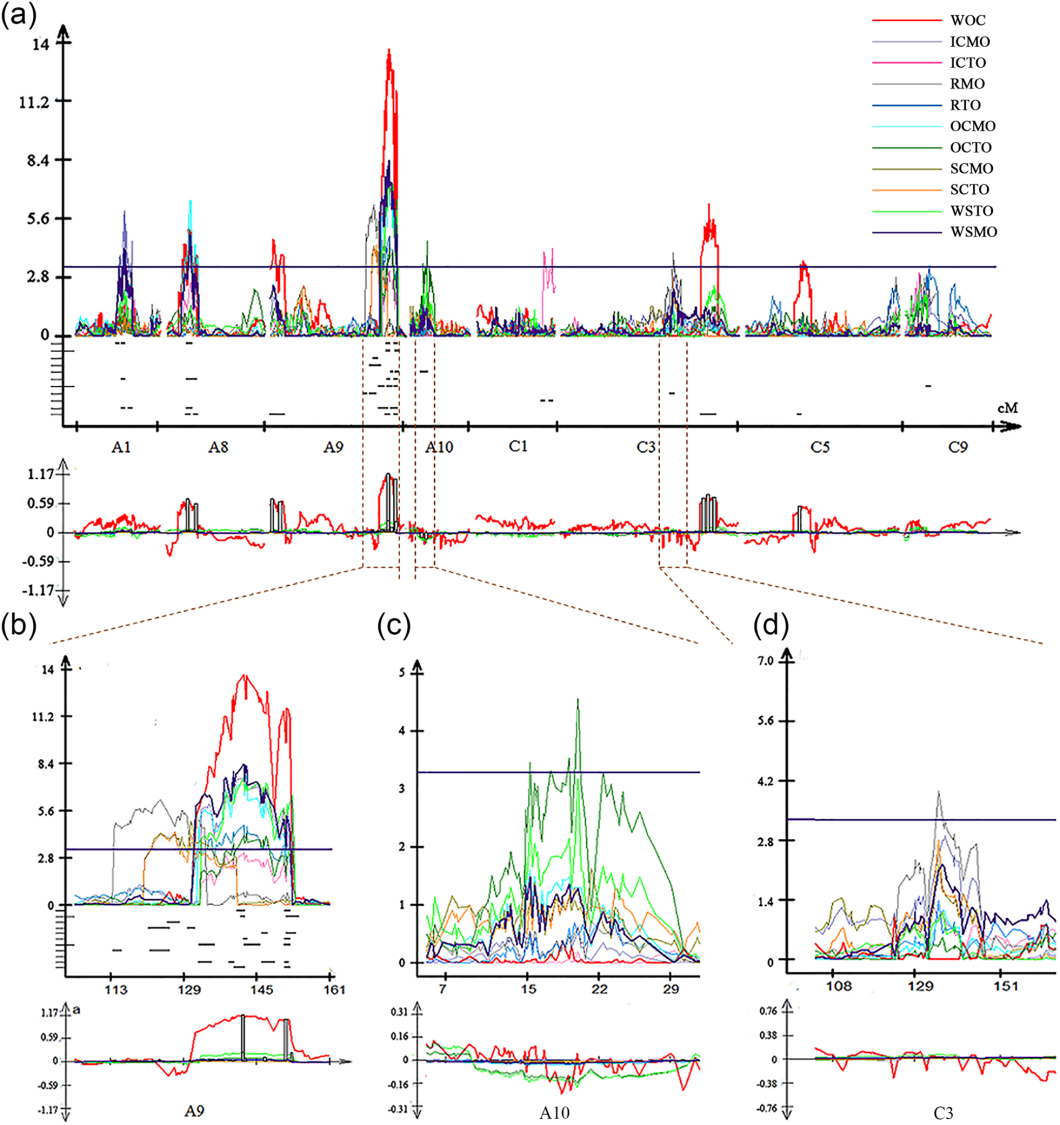
QTLs for oil content in different tissues of seeds in the KN DH population. **a** Distribution of identified QTLs for oil content in different tissues of seeds in each linkage group. **b**, **c**, and **d** represent the distribution of common and specific QTLs for the oil content of different seed tissues in the A9, A10 and C3 linkage groups, respectively. WOC refers to identified QTLs for relative oil content detected by near-infrared spectroscopy

QTL mapping for the ten oil content-related traits (OCTO, ICTO, RTO, SCTO, OCMO, ICMO, RMO, SCMO, WSTO and WSMO) was implemented based on the above phenotyping data and by combining a high-density genetic map containing 3207 markers [62]. A total of 35 QTLs, which could explain 5.22–13.76% of the phenotypic variation (PV), were identified and mainly distributed on chromosomes A1, A8, A9, A10, C1, C3 and C9 (Table 1; Fig. 4a; Additional file 1: Fig. S4). Thirteen QTLs were identified for TO, of which 2 QTLs for WSTO (*qWSTO-A9-1* and *qWSTO-A9-2*) on the A9 chromosome had a PV of more than 10% and had a relatively large additive effect (> 0.2) (Table 1; Fig. 4a). In addition, 4, 2, 4 and 1 for OCTO, ICTO, RTO and SCTO, respectively, were located on A9 with relatively lower PV and additive effect (Table 1; Fig. 4b). Five, five, seven, three and two QTLs were identified for MO of the whole seed and subdivided tissues (WSMO, OCMO, ICMO, RMO and SCMO) and were distributed on the chromosomes of A1, A8, A9 and C3. Most MO-QTLs (12 of 22) were distributed on A9 (Fig. 4b).

**Table 1:** Identified QTLs and unique QTLs identified by high-density linkage map for oil content in different tissues of seeds in the KN DH population

Tissues-QTL														KN^a^		KN^b^ and TN^c^ populations	
u-QTL	LOD	Additive	PV	QTL	Chr^a^	LOD	Additive	PV	Position	Range-L	Range-R	Chr^a^. Region-L (Mb)a	Chr^a^. Region-R (Mb)	Consensus QTL	Range	QTL	Chr. Region (Mb)
*uqA1-1*	3.31	0.031	5.22	*qWSMO-A1-1*	A1	3.31	0.031	5.22	52.51	49.8	53.5	7.05	7.91				
*uqA1-2*	3.70–5.50	0.036–0.045	5.88–9.21	*qICMO-A1-1*	A1	5.51	0.045	9.21	58.91	58.9	60	14.04	15.55				
				*qOCMO-A1-2*	A1	3.70	0.044	5.88	58.91	58	60						
				*qWSMO-A1-2*	A1	3.81	0.036	6.07	58.91	58.9	60						
*uqA1-3*	4.56	0.039	7.60	*qICMO-A1-2*	A1	4.56	0.039	7.60	67.81	67	68.9	18.22	18.65				
*uqA8-1*	3.92–6.51	0.034–0.053	6.25–10.37	*qICMO-A8-1*	A8	3.92	0.034	6.25	27.61	25.9	28.8	8.59	10.84	*cqOC-A8-3*	24.7–27.6	*KN-qOC-A8-2*	9.35–12.03
				*qOCMO-A8-1*	A8	6.51	0.053	10.37	27.61	25.9	29.7						
				*qWSMO-A8-1*	A8	4.86	0.037	7.63	27.61	24.3	30						
*uqA8-2*	4.40	0.044	7.18	*qOCMO-A8-2*	A8	4.40	0.044	7.18	33.31	31.7	34.6	10.78	11.07			*KN-qOC-A8-2*	9.35–12.03
*uqA9-1*	4.92	0.034	8.57	*qRMO-A9-1*	A9	4.92	0.034	8.57	113.91	113.7	115.2	26.06	26.46			*TN-qOC-A9-3*	24.48–27.13
*uqA9-2*	4.31–6.26	0.023–0.038	8.19–10.75	*qRMO-A9-2*	A9	6.26	0.038	10.75	123.81	121.9	127	27.06	28.06			*TN-qOC-A9-3*	24.48–27.13
				*qSCMO-A9-2*	A9	4.31	0.024	8.19	123.81	120.3	125.8						
*uqA9-3*	4.39	0.012	8.09	*qSCTO-A9-2*	A9	4.39	0.012	8.09	127.01	125.8	128.1	27.92	28.25				
*uqA9-4*	5.00	0.025	9.45	*qSCMO-A9-3*	A9	5.00	0.025	9.45	130.51	129.8	131.6	28.31	28.49				
*uqA9-5*	3.94–6.02	0.016–0.046	7.15–10.19	*qICMO-A9-1*	A9	6.02	0.046	10.19	134.51	132.6	135.6	28.49	29.72				
				*qRTO-A9-1*	A9	3.94	0.016	7.15	134.91	132.7	135.6						
*uqA9-6*	7.57	0.052	12.63	*qICMO-A9-2*	A9	7.57	0.052	12.63	139.81	137.7	139.9	29.99	30.61			*TN-qOC-A9-4*	29.49–31.23
*uqA9-7*	7.61–8.39	0.052–0.224	13.46–13.76	*qWSTO-A9-1*	A9	7.61	0.224	13.46	142.11	139.9	142.3	30.50	30.82	*cqOC-A9-5*	140.87–142.35	*TN-qOC-A9-4*	29.49–31.23
				*qWSMO-A9-2*	A9	8.39	0.052	13.76	142.11	139.9	142.3						
*uqA9-8*	7.74	0.062	12.52	*qOCMO-A9-2*	A9	7.74	0.062	12.52	142.61	142.3	142.9	30.68	31.17			*TN-qOC-A9-4*	29.49–31.23
*uqA9-9*	4.69	0.017	8.50	*qRTO-A9-2*	A9	4.69	0.017	8.50	142.81	142.6	145.9	30.68	31.17			*TN-qOC-A9-4*	29.49–31.23
*uqA9-10*	4.14–6.67	0.106–0.048	7.23–11.27	*qOCTO-A9-1*	A9	4.14	0.106	7.23	146.91	146.4	147.2	31.21	32.53	*cqOC-A9-7*	146.89–147.59		
				*qICMO-A9-3*	A9	6.67	0.048	11.27	147.21	146.4	148.8						
*uqA9-11*	3.70–6.02	0.015–0.046	6.71–10.19	*qRTO-A9-3*	A9	3.70	0.015	6.71	151.51	151.3	152.3	32.53	32.82				
				*qOCMO-A9-3*	A9	4.47	0.046	7.51	151.51	151.3	152.1						
				*qWSMO-A9-3*	A9	5.33	0.041	9.04	151.51	151.3	152.1						
				*qICMO-A9-4*	A9	6.02	0.045	10.19	151.51	151.3	152.1						
*uqA9-12*	3.48–6.54	0.098–0.208	6.12–11.74	*qOCTO-A9-2*	A9	3.48	0.098	6.12	152.71	151.8	154.1	32.79	33.75	*cqOC-A9-9*	152.34–153.03		
				*qWSTO-A9-2*	A9	6.54	0.208	11.74	152.71	151.8	155.2						
*uqA10-1*	3.47	− 0.120	6.17	*qOCTO-A10-1*	A10	3.47	-0.120	6.17	15.21	14.9	15.4	8.17	8.78			*TN-qOC-A10*	6.25–11.00
*uqA10-2*	4.57	− 0.153	8.07	*qOCTO-A10-2*	A10	4.57	-0.153	8.07	19.91	19.4	20.6	11.30	11.81			*KN-qOC-A10*	9.00–14.08
*uqC1-1*	4.04	− 0.050	7.08	*qICTO-C1*	C1	4.04	-0.050	7.08	80.51	79.6	80.9	32.45	34.41				
*uqC1-2*	4.19	− 0.050	7.44	*qICTO-C2*	C1	4.19	-0.050	7.44	90.61	89.4	91.2	36.21	36.39				
*uqC3*	4.00	0.030	6.71	*qRMO-C3-1*	C3	4.00	0.030	6.71	134.91	132.7	136.4	28.13	32.84				
*uqC9*	3.38	0.014	5.97	*qRTO-C9-2*	C9	3.38	0.014	5.97	29.91	28	31	6.48	13.03				

^a^QTL identified in the research Chao et al. reported [62]

^b^and ^c^represent QTLs identified in KN and TN populations that Wang et al. [49] and Jiang et al. [28] reported, respectively

The above 35 QTLs were integrated into 23 unique QTLs by meta-analysis (Table 1), and 8 unique QTLs had pleiotropic effects. Four (*uqA9-5*, *uqA9-7*, *uqA9-10* and *uqA9-11*) were responsible for MO and TO simultaneously and were all located on A9, and four unique QTLs (*uqA1-2*, *uqA8-1*, *uqA9-2*, and *uqA9-12*) were responsible for MO or TO of the same trait and were located on A1, A8, A9 and A9, respectively (Table 1). *uqA1-2*, *uqA8-1* and *uqA9-11* affected the OCMO, ICMO and WSMO simultaneously, which was consistent with the phenotypic performances that OC and IC had the largest effect on WSMO. Further analysis revealed that the pleiotropic QTLs *uqA8-1*, *uqA9-7*, *uqA9-10* and *uqA9-12* that responded to IC, OC and WS were also identified in our previous studies based on the oil content of whole seeds [62] (Table 1). Furthermore, *uqA9-11*, which was identified to control RTO, OCMO and ICMO simultaneously, was a novel locus with pleiotropic effects on the oil content of different tissues.

**Fig. 5:**
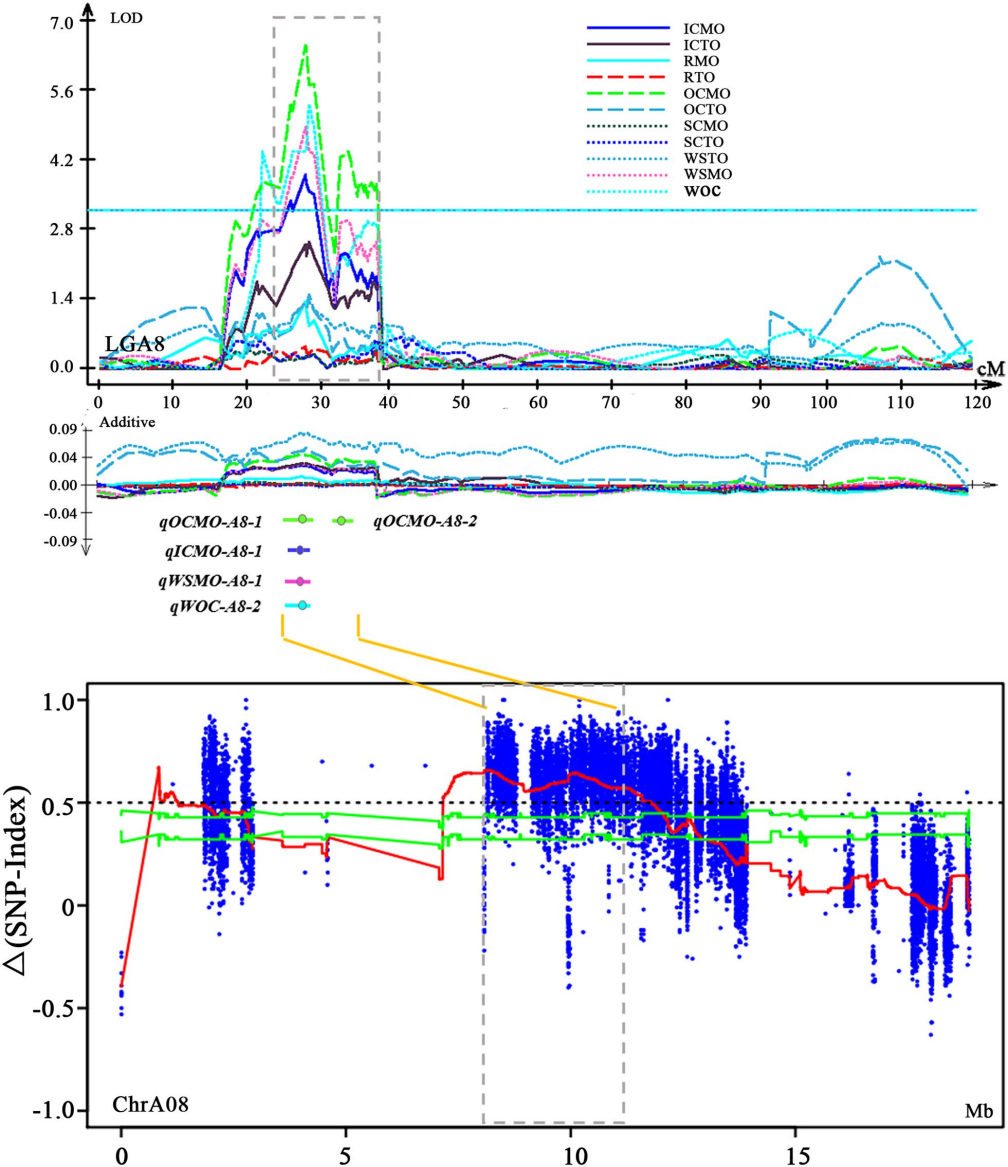
*uqA8-1* controlling OCMO, ICMO, and WSMO was also identified on BSA. Original QTL identification shown by curves above the line of the linkage group (top). The Δ (SNP index) plot with statistically significantly associated regions (two green significant threshold lines, *P* < 0.01 and *P* < 0.05) is drawn at the bottom. In the two coordinate axes, the X-axis represents the position of linkage group A8 and chromosome A08, and the Y-axis represents the LOD value and Δ (SNP index). The additive effect was also shown under linkage Group A8. In the middle, the CI and peak of *qOCMO-A8-1*, *qICMO-A8-1*, *qWSMO-A8-1, qOCMO-A8-2* and *qWOC-A8-2* are shown. WOC represents QTLs identified for relative oil content. The middle connecting line links QTLs and their corresponding AGRs

Fourteen unique QTLs that were identified to only control MO and/or TO in one tissue were considered to be tissue-specific QTLs and would provide new potential loci or genes for breeding to improve seed oil content (Table 1; Fig. 4a). Furthermore, *uqA9-1* and *uqA9-9*, which were two adjacent QTLs, were identified for MO and TO of the R, respectively, while *uqA9-3* and *uqA9-4* were identified for TO and MO of the SC, respectively (Fig. 4b); *uqA1-3* and *uqA9-6* were identified for MO of the IC, while *uqA8-2* and *uqA9-8* were identified for MO of the OC. In addition, both *uqC1-1* and *uqC1-2* controlled the TO of the IC, while *uqA10-1* and *uqA10-2* were identified to be specific for TO of the OC (Fig. 4c)*; uqC3* and *uqC9* were specific for RMO and RTO, respectively (Fig. 4a and d). All these QTLs were thought to have some effect on oil accumulation in corresponding tissues of the seeds, and these tissue-specific QTLs were the first reported in *B. napus*. Moreover, combined with BSA results for oil content reported in our previous study [62], it was revealed that many common significant variation loci revealed by BSA analysis were also identified in the present study (15 QTLs, most of them for OC and IC) (Table 2). For instance, *uqA8-1* controlling the OCMO, ICMO and WSMO was identified in BSA analysis and colocalized with a QTL (*cqOC-A8-3*) identified for oil content of the whole seed (Fig. 5; Table 2).

**Table 2:** AGRs overlapping with QTLs for oil content from the QTL mapping method based on a high-density genetic linkage map

Tissue-specific QTL mapping				BSA			QTL mapping based on relative oil content		
QTL	Chr	Genetic distance (cM)	Physic distance (Mb)	Associated genomic region	Chr	Physic distance (Mb)	QTL	Chr	Physic distance (Mb)
*qICMO-A8-1*	A8	25.9–28.8	8.59–10.84	AGR_A8-4	chrA08	7.15–13.07	cqOC-A8-3	A08	9.35–12.03
*qOCMO-A8-1*	A8	25.9–29.7							
*qWSMO-A8-1*	A8	24.3–30							
*qOCMO-A8-2*	A8	31.70–34.60	10.79–11.08						
*qICMO-A9-2*	A9	137.7–139.9	30–30.62	AGR_A9-8	chrA09	29.78–31.26	cqOC-A9-5	A09	29.49–31.23
*qWSTO-A9-1*	A9	139.9–142.3	30.5–30.82						
*qWSMO-A9-2*	A9	139.9–142.3	30.5–30.82						
*qOCTO-A9-1*	A9	146.4–147.2	31.21–32.54	AGR_A9-9	chrA09	31.29–31.30	cqOC-A9-7	A09	31.09—33.75
*qICMO-A9-3*	A9	146.4–148.8		AGR_A9-11	chrA09	31.52–31.61			
*qRTO-A9-3*	A9	151.3–152.1	32.54–32.83	AGR_A9-12	chrA09	31.62–33.86			
*qOCMO-A9-3*	A9	151.3–152.3					cqOC-A9-9	A09	33.75—33.83
*qWSMO-A9-3*	A9	151.3–152.1							
*qICMO-A9-4*	A9	151.3–152.1							
*qOCTO-A9-2*	A9	151.8–154.1	32.79–33.75						
*qWSTO-A9-2*	A9	151.8–155.2

### Favorable alleles for oil content from different tissues exhibit cumulative effects

**Fig. 6:**
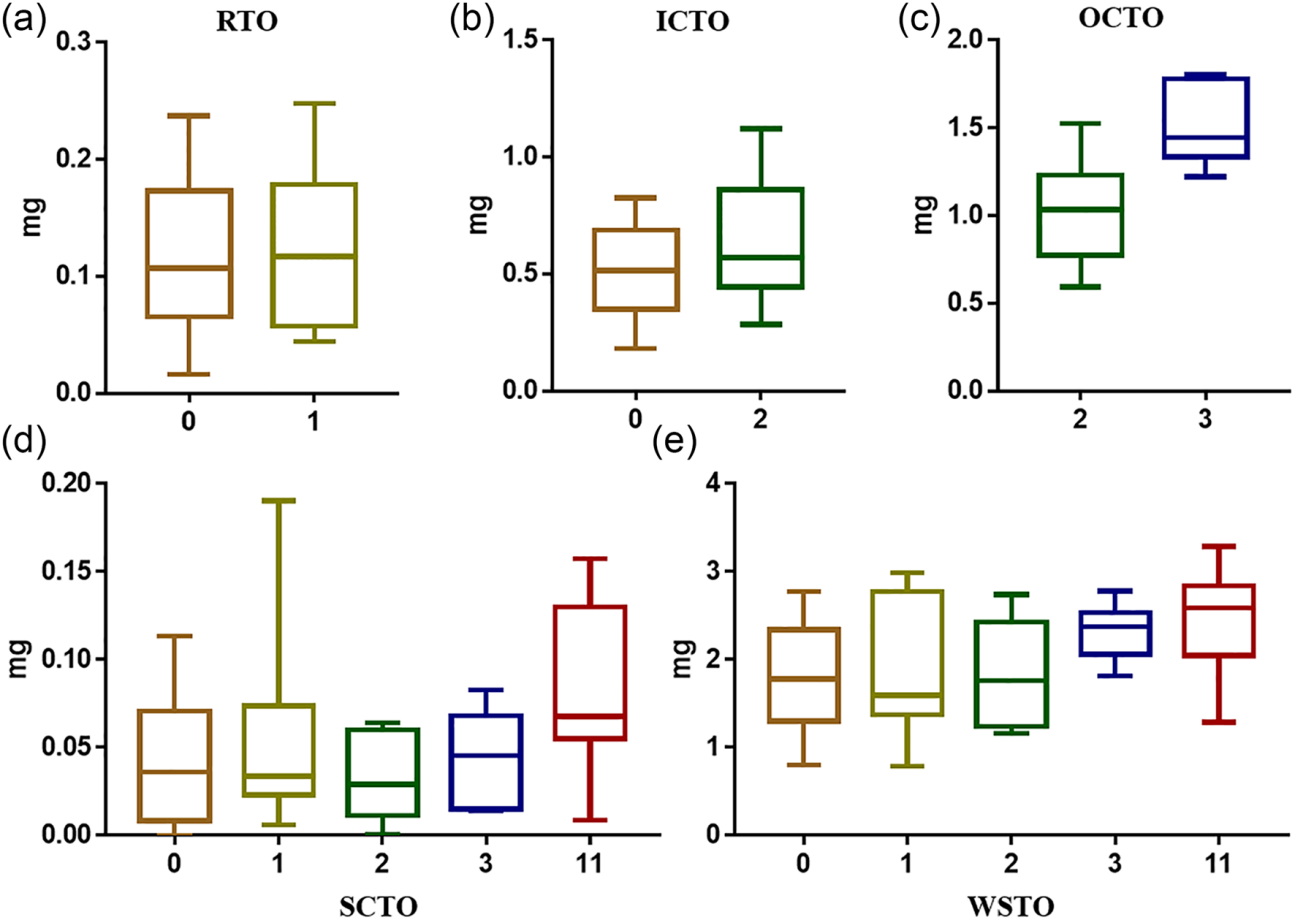
Cumulative effects of tissue-specific TO-QTLs. The numbers on the ordinate represent the number of accumulating QTLs with favorable haplotypes. 0 represents the phenotype value of DH lines with undesirable haplotypes at all 12 assessed TO-QTLs. 1 represents DH lines with a favorable haplotype in only one QTL (*qRTO-C9-2*). 2 represents DH lines with favorable haplotypes in two QTLs (*qICTO-C1-1* and *qICTO-C1-2*). 3 represents DH lines with favorable haplotypes in three QTLs (*qICTO-C1-1*, *qICTO-C1-2* and *qOCTO-A10-2*). 11 represents those DH lines that harbored 11 QTLs with favorable haplotypes involved in SCTO, RTO, ICTO and OCTO. **a**, **b**, **c** and **d** show the phenotypic values of RTO, ICTO, OCTO and SCTO variation in DH lines with different types and numbers of favorable alleles, respectively. **e** shows the WSTO variation along with the accumulation of different types and numbers of favorable alleles

To understand the effects of QTLs for different tissues on the oil content of the whole seed, allelic genotypes in TO-QTLs and phenotypes for different tissues and the whole seed were assessed based on DH lines from the KN population (Additional file 2: Table S3). The results revealed that the TO of tissues (IC, OC and SC) and WSTO gradually increased with an increasing number of favorable haplotypes (Fig. 6). DH lines with favorable haplotypes in three QTLs, including two ICTO-QTLs (*qICTO-C1-1* and *qICTO-C1-2*) and one OCTO-QTL (*qOCTO-A10-2*), showed higher OCTO and WSTO than DH lines without favorable haplotypes and DH lines with favorable haplotypes in only two ICTO-QTLs (*qICTO-C1-1 and qICTO-C1-2*) (Fig. 6b, c). In addition, the SCTO changed insignificantly when QTLs with favorable haplotypes involved in RTO, ICTO and OCTO aggregated, but those DH lines that harbored 11 QTLs with favorable haplotypes involved in SCTO, RTO, ICTO and OCTO showed a higher SCTO (Fig. 6d). Most importantly, more QTL accumulation caused a significant increase in WSTO, especially when OCTO-QTLs were affiliated (Fig. 6e). These results suggested that the genetic control of the seed oil content in *B. napus* exhibits an additive effect, and accumulation of OCTO played a key role in the improvement of total seed oil content.

### Characteristics of gene expression in different seed tissues

A total of 48 RNA samples from four seed tissues (SC, R, IC and OC) of Ken-C8 (with lower oil content) and N53-2 (with higher oil content) at 24 and 33 DAF (with three biological replicates) were collected for transcriptome sequencing, and 2675 million clean reads, with an average of 55.72 million reads per sample, were generated (Additional file 2: Table S4). The total mapped reads ranged from 35.08 to 66.27 million reads, with 61.00 million reads per sample on average, and the average unique mapping rate was 86.00%. All 48 samples showed high reproducibility (*r*^2^ > 0.9) among biological replicates in different tissues of Ken-C8 and N53-2 (Additional file 1: Fig. S5).

**Fig. 7:**
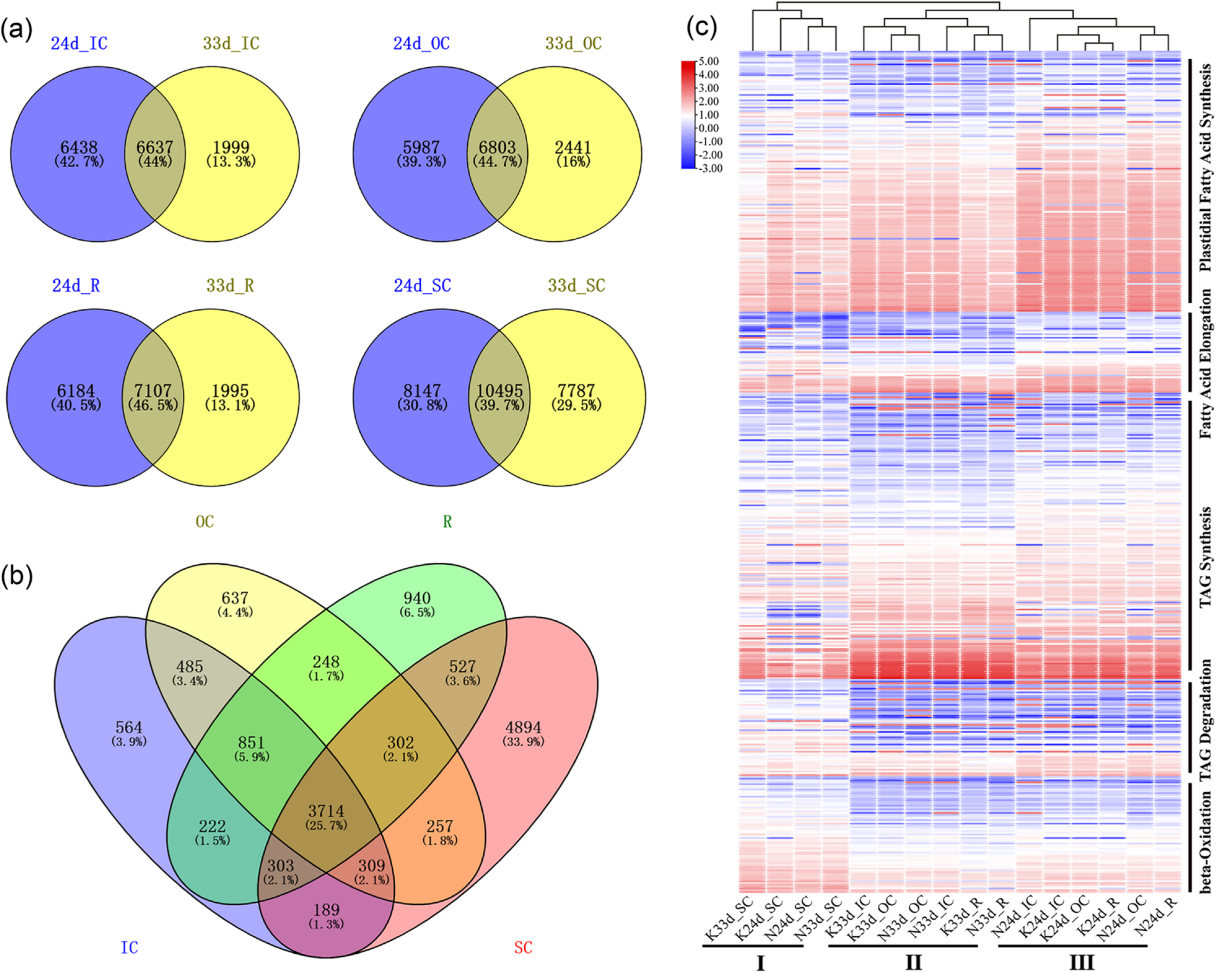
Differentially expressed gene analysis. **a** Venn diagrams show the common and different DEGs (Ken-C8 vs. N53-2) in the four tissues. **b** Venn diagrams show the common and different DEGs among different tissues at 24 and/or 33 DAP. **c** Clustering analysis based on the expression level of the DEGs involved in the TAG biosynthesis pathway, including plastidial fatty acid synthesis, fatty acid elongation, TAG synthesis, TAG degradation and beta-oxidation, among different samples

By comparing the 4 tissues at the 2 stages between Ken-C8 and N53-2, 15,064, 15,231, 15,286 and 26,429 DEGs were identified in IC, OC, R and SC, respectively. Further analysis revealed that 6637, 6083, 7107 and 10495 DEGs existed at 24 and 33 DAF in IC, OC, R and SC, respectively (Fig. 7a, b). Further analysis revealed that the IC, OC and R were clustered together instead of different lines and developmental stages together for each tissue based on the expression level of DEGs (Additional file 1: Fig. S6), which indicated that the IC, OC and R were highly similar and different from SC from the perspective of gene expression. In addition, the KEGG enrichment analysis for tissue-specific DEGs (Ken-C8 vs. N53-2) at 24 and 33 DAF in the IC, OC, R and SC showed that pyruvate metabolism was significantly enriched in IC, OC and R (Additional file 1: Fig. S7), which indicated a relatively greater tendency toward more pyruvate utilization for the accumulation of major compounds, including oil, protein and other carbohydrates, in these three tissues.

Furthermore, the DEGs involved in triacylglycerol (TAG) biosynthesis (including plastidial fatty acid (FA) synthesis, fatty acid elongation, TAG synthesis, TAG degradation and beta-oxidation) among different tissues were identified, and cluster analysis showed that the tissue samples could be divided into three distinct clusters: four SC samples from Ken-C8 and N53-2 collected at 24 and 33 DAF were clustered together (I), and samples of IC, OC and R collected at 33 (II) and 24 (III) DAF were clustered together (Fig. 7c). DEGs with relatively higher expression levels involved in plastidial fatty acid synthesis were found in cluster III, which indicated that fatty acid synthesis was more active in IC, OC and R at 24 DAF. For example, *BnaC09. BCCP2* (the subunit of heteromeric ACCase) and *BnaA01. LPD2* (the E3 component of the pyruvate dehydrogenase complex) showed higher expression levels in IC, OC and R at 24 DAF than at 33 DAF (Additional file 1: Fig. S8). Otherwise, genes involved in TAG synthesis with higher expression levels were also found in cluster II, which indicated that TAG synthesis was more active in IC, OC and R at 33 DAF, such as *BnaA09g02110D* and *BnaC04g32530D* (both encode the oil-body oleosin), which were more highly expressed in IC, OC and R at 33 DAF than at 24 DAF (Additional file 1: Fig. S8). In contrast, the β-oxidation of TAG tended to be more active in SC at 24 and 33 DAF (Additional file 1: Fig. S8).

**Fig. 8:**
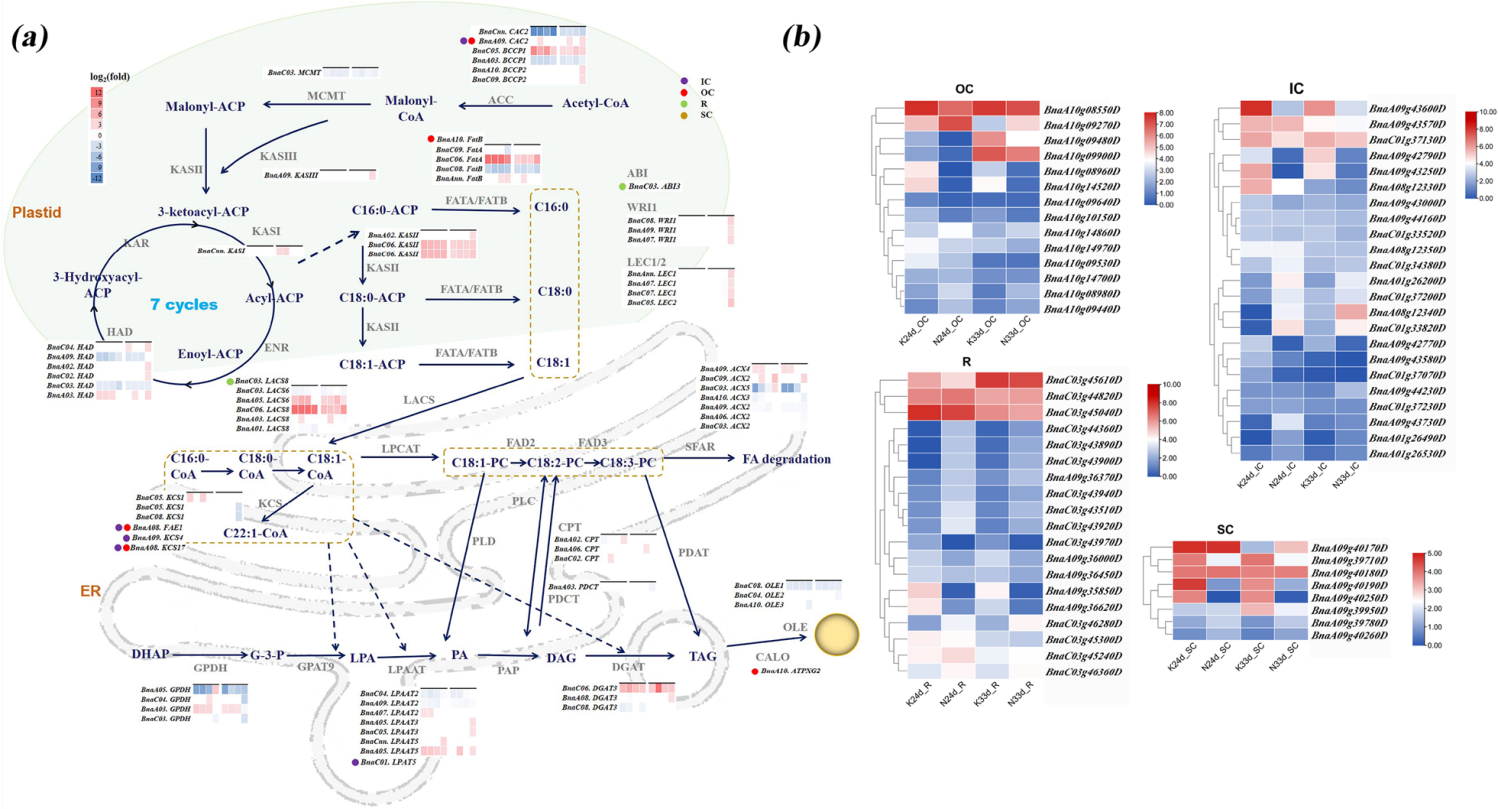
TAG biosynthesis and accumulation-related pathways regulating the oil content. Gene expression differences between the two parents are shown in the pathway, and homologous copies showing differential expression in any tissue are listed next to the protein. The 24 and 33 DAP were defined by horizontal lines, and the positions of colored squares below the horizontal line are as follows: IC, OC, R and SC. The genes located in the QTL interval are marked with colored dots, and the color annotation is located in the top right corner. The expression difference of DEGs identified in the QTL interval for the four tissues

To more intuitively understand the differences in the transcriptional regulation of lipid formation in IC, OC, R and SC between Ken-C8 and N53-2, a TAG biosynthesis- and accumulation-related pathway was reconstructed by integrating differential gene expression in different tissues according to RNA-Seq (Fig. 8). *BnaC06*. *FatA* and *BnaC06.* *KASII* were differentially expressed in all four tissues, and higher expression in N53-2 with higher oil content was observed. These key genes involved in the FA synthetic pathway showed significantly different expression levels in the four tissues, which suggested that various tissues of seeds in N53-2 have more FA synthesis activity than those of Ken-C8. The genes involved in the TAG synthetic pathway, such as *BnaA03. GPDH*, *BnaA03. LPAAT5* and *BnaC06. DGAT3* showed higher expression levels in the four tissues of N53-2, especially in IC and OC, which suggested a difference in TAG synthesis ability in different tissues between the two parents with high and low seed oil content. Interestingly, three copies of two important transcription factors, *WRI1* and *LEC1*, were found to have significantly different expression only in the SC at 33 DAP, which suggested that the SC of N53-2 maintained a higher FA synthesis ability than that of Ken-C8 at the late stage of seed development, which was verified by the higher expression level of *BnaC09. CAC2*, *BnaA02. HAD*, *BnaC02. HAD* and *BnaA02. KASII,* which is regulated by *WRI1* and *LEC1* (Fig. 8a). This phenomenon also indicated that the high fatty acid synthesis activity of the tissue SC is still maintained in the late stage of seed development, which may be an important factor for the formation of high oil content.

### Identifying candidate genes for oil content QTLs in subdivided seed tissues

The candidate genes within QTL intervals were screened according to the relationship between the genetic map and the physical map. Overall, 861 DEGs between Ken-C8 and N53-2 at 24 and 33 DAF were found to be located in the interval of QTLs for different seed tissues. Fourteen of OC, 23 of IC, 19 of R and 8 of SC were located in the genomic region of QTL for IC, OC, R and SC, respectively (Fig. 8b). For example, *BnaA09g43600D*, which was highly expressed in the IC of Ken-C8 at 24 and 33 DAF, was located in the QTL *qICMO-A9-2*. *BnaA10g09270D*, which is located in the OC-specific QTL *qOCTO-A10-1*, showed higher expression in the OC of N53-2 at 24 and 33 DAF. *BnaA09g35850D* was differentially expressed in R at 24 and 33 DAF, and it was identified as underlying the R-QTL *qRMO-A9-1*. *BnaA09g39710D* (located in *qSCTO-A9-2*), *BnaA09g40190D* and *BnaA09g40250D* (located in *qSCTO-A9-3*) showed higher expression in the SC of Ken-C8 at 24 and 33 DAF.

Through annotation from the *A. thaliana* database (TAIR: http://www.arabidopsis.org/), 86 candidate genes within intervals of 19 unique QTLs were identified to be associated with lipid metabolism (Table 3). Three of these related genes (*CAC2*, *KCS17* and *FAE1*) that are directly involved in FA synthesis and TAG metabolism were identified as pleiotropic genes for OC and IC. *BnaA09g48250D*, which is within *uqA9-10* and affects OCTO and ICMO, was annotated as *CAC2* (*BnaA09. CAC2*), the biotin carboxylase (BC) domain of *ACC,* which catalyzes the first committed step in fatty acid synthesis. *BnaA09. CAC2* was significantly more highly expressed in the OC of N53-2 at 24 and 33 DAF compared to Ken-C8 (Fig. 8). *BnaA08g11140D* (homologous to *AtKCS17*) and *BnaA08g11130D* (homologous to *AtFAE1*), which mapped within the CI of *uqA8-1*, simultaneously control ICMO, OCMO and WSMO and encode 3-ketoacyl-CoA synthases involved in the biosynthesis of very long-chain fatty acids. It was exciting that as many as ten candidate genes involved in phospholipase, aliphatic suberin synthesis, lipase, sphingolipid synthesis, fatty acid elongation and cuticular wax synthesis were found to be located in CI of *uqA8-1*, of which *BnaA08g10850D* encoding phospholipase showed different expression in IC between N53-2 and Ken-C8. In addition, differential expression of *BnaA09g38540D*, *BnaA09g38630D* and *BnaA09g39290D* (homologous to *AtG3*, *AtCDS5* and *AT3G61580*, located in the overlapping CI of *qRMO-A9-2* and *qSCMO-A9-2*), which are involved in cuticular wax synthesis, sulfolipid synthesis and sphingolipid synthesis, was observed in all tissues.

**Table 3:** Identification of candidate genes for oil content QTLs in different seed tissues in the KN DH population

Unique-QTL	QTL	Gene alias	*A. thaliana* Locus	ARALIP pathway(s)	Gene symbol	ARALIP protein family name/isoform
*uqA1-1*	*qWSMO-A1-1*	*BnaA01g14400D*	*AT4G25050*	Plastidial fatty acid synthesis	*ACP4*	Acyl carrier protein
*uqA9-6*	*qICMO-A9-2*	*BnaA09g42770D*	*AT2G22230*	Plastidial fatty acid synthesis		Hydroxyacyl-ACP dehydrase
*uqA9-10*	*qOCTO-A9-1* + *qICMO-A9-3*	*BnaA09g48250D*	*AT5G35360*	Plastidial fatty acid synthesis	*CAC2*	Biotin carboxylase of heteromeric ACCase
*uqA10-1*	*qOCTO-A10-1*	*BnaA10g09300D*	*AT1G08510*	Plastidial fatty acid synthesis	*FATB*	Acyl-ACP thioesterase B
*uqC3*	*qRMO-C3-1*	*BnaC03g44430D*	*AT2G04350*	Plastidial fatty acid synthesis	*LACS8*	Long-chain acyl-CoA synthetase (plastidial); long-chain acyl-CoA synthetase
*uqC3*	*qRMO-C3-1*	*BnaC03g45040D*	*AT2G05990*	Plastidial fatty acid synthesis	*MOD1*	Enoyl-ACP reductase
*uqA8-1*	*qICMO-A8-1* + *qOCMO-A8-1* + *qWSMO-A8-1*	*BnaA08g11140D*	*AT4G34510*	Fatty acid elongation and cuticular wax synthesis	*KCS17*	Ketoacyl-CoA synthase
*uqA8-1*	*qICMO-A8-1* + *qOCMO-A8-1* + *qWSMO-A8-1*	*BnaA08g11130D*	*AT4G34520*	Fatty acid elongation and cuticular wax synthesis	*FAE1*	Ketoacyl-CoA synthase
*uqA9-5*	*qICMO-A9-1* + *qRTO-A9-1*	*BnaA09g40640D*	*AT2G26250*	Fatty acid elongation and cuticular wax synthesis	*KCS10*	Ketoacyl-CoA synthase
*uqA9-6*	*qICMO-A9-2*	*BnaA09g44160D*	*AT1G19440*	Fatty acid elongation and cuticular wax synthesis	*KCS4*	Ketoacyl-CoA synthase
*uqC3*	*qRMO-C3-1*	*BnaC03g46130D*	*AT2G16280*	Fatty acid elongation and cuticular wax synthesis	*KCS9*	Ketoacyl-CoA synthase
*uqA9-4*	*qSCMO-A9-3*	*BnaA09g40180D*	*AT3G62860*	TAG degradation		Monoacylglycerol lipase (MAGL)
*uqA1-1*	*qWSMO-A1-1*	*BnaA01g14480D*	*AT4G25140*	TAG synthesis	*OLEO1*	Oil-body oleosin
*uqA10-1*	*qOCTO-A10-1*	*BnaA10g09480D*	*AT5G55240*	TAG synthesis	*ATPXG2*	Caleosin
*uqC1-1*	*qICTO-C1-1*	*BnaC01g34070D*	*AT3G18570*	TAG synthesis		Oil-body oleosin
*uqC1-1*	*qICTO-C1-1*	*BnaC01g33800D*	*AT3G18850*	TAG synthesis	*LPAT5*	1-Acylglycerol-3-phosphate acyltransferase; 1-acylglycerol-3-phosphate acyltransferase
*uqC3*	*qRMO-C3-1*	*BnaC03g44820D*	*AT3G24650*	TAG synthesis	*ABI3*	Homologous to the maize transcription factor Viviparous-1
*uqA9-2*	*qRMO-A9-2* + *qSCMO-A9-2*	*BnaA09g38630D*	*AT3G60620*	Plastidial glycerolipid, galactolipid and sulfolipid synthesis	*CDS5*	CDP-DAG Synthase (plastidial)
*uqA9-9*	*qRTO-A9-2*	*BnaA09g45250D*	*AT1G15080*	Plastidial glycerolipid, galactolipid and sulfolipid synthesis	*LPP2*	Phosphatidate phosphatase
*uqA8-1*	*qICMO-A8-1* + *qOCMO-A8-1* + *qWSMO-A8-1*	*BnaA08g10960D*	*AT4G34930*	Phospholipase		Glycosylphosphatidylinositol-specific phospholipase C
*uqA8-1*	*qICMO-A8-1* + *qOCMO-A8-1* + *qWSMO-A8-1*	*BnaA08g10850D*	*AT4G35110*	Phospholipase		
*uqA9-6*	*qICMO-A9-2*	*BnaA09g43510D*	*AT2G20950*	Phospholipase		
*uqA9-6*	*qICMO-A9-2*	*BnaA09g43500D*	*AT2G20960*	Phospholipase	*pEARLI4*	
*uqC3*	*qRMO-C3-1*	*BnaC03g46730D*	*AT2G16900*	Phospholipase		
*uqA8-1*	*qICMO-A8-1* + *qOCMO-A8-1* + *qWSMO-A8-1*	*BnaA08g11440D*	*AT4G33790*	Aliphatic suberin synthesis	*CER4*	Alcohol-forming fatty acyl-CoA reductase (ER); alcohol-forming fatty acyl-CoA reductase
*uqA9-10*	*qOCTO-A9-1* + *qICMO-A9-3*	*BnaA09g45720D*	*AT1G14190*	Aliphatic suberin synthesis		Omega-hydroxy fatty acyl dehydrogenase; omega-hydroxy fatty acyl dehydrogenase
*uqA8-1*	*qICMO-A8-1* + *qOCMO-A8-1* + *qWSMO-A8-1*	*BnaA08g11640D*	*AT4G34050*	Aromatic suberin synthesis	*CCoAOMT1*	Caffeoyl-CoA O-methyltransferase
*uqA1-1*	*qWSMO-A1-1*	*BnaA01g14970D*	*AT4G25750*	Cuticular wax synthesis		ABC transporter
*uqA8-1*	*qICMO-A8-1* + *qOCMO-A8-1* + *qWSMO-A8-1*	*BnaA08g10330D*	*AT4G22520*	Cuticular wax synthesis		Lipid transfer protein type 6
*uqA8-1*	*qICMO-A8-1* + *qOCMO-A8-1* + *qWSMO-A8-1*	*BnaA08g11810D*	*AT4G33355*	Cuticular wax synthesis		Lipid transfer protein type 1
*uqA9-2*	*qRMO-A9-2* + *qSCMO-A9-2*	*BnaA09g38540D*	*AT3G60500*	Cuticular wax synthesis	*G3*	CER7 protein involved in wax synthesis
*uqA9-5*	*qICMO-A9-1* + *qRTO-A9-1*	*BnaA09g42120D*	*AT2G23180*	Cuticular wax synthesis	*CYP96A1*	Midchain alkane hydroxylase / cytochrome P450, 96A
*uqA9-7*	*qWSTO-A9-1* + *qWSMO-A9-2*	*BnaA09g44630D*	*AT1G18280*	Cuticular wax synthesis		Lipid transfer protein type 5
*uqA10-1*	*qOCTO-A10-1*	*BnaA10g09280D*	*AT3G53510*	Cuticular wax synthesis		ABC transporter
*uqA10-1*	*qOCTO-A10-1*	*BnaA10g09530D*	*AT5G55340*	Cuticular wax synthesis		Wax synthase
*uqA10-1*	*qOCTO-A10-1*	*BnaA10g09620D*	*AT5G55350*	Cuticular wax synthesis		Wax synthase
*uqA10-1*	*qOCTO-A10-1*	*BnaA10g09630D*	*AT5G55360*	Cuticular wax synthesis		Wax synthase
*uqA10-1*	*qOCTO-A10-1*	*BnaA10g09610D*	*AT5G55380*	Cuticular wax synthesis		Wax synthase
*uqA10-1*	*qOCTO-A10-1*	*BnaA10g09640D*	*AT5G55410*	Cuticular wax synthesis		Lipid transfer protein type 3
*uqA10-1*	*qOCTO-A10-1*	*BnaA10g09590D*	*AT5G55460*	Cuticular wax synthesis		Lipid transfer protein type 3
*uqC1-1*	*qICTO-C1-1*	*BnaC01g34380D*	*AT3G18280*	Cuticular wax synthesis		Lipid transfer protein type 2
*uqC3*	*qRMO-C3-1*	*BnaC03g45240D*	*AT2G13610*	Cuticular wax synthesis		ABC transporter
*uqC3*	*qRMO-C3-1*	*BnaC03g45300D*	*AT2G13820*	Cuticular wax synthesis		Lipid transfer protein type 5
*uqA1-1*	*qWSMO-A1-1*	*BnaA01g15140D*	*AT4G25970*	Eukaryotic phospholipid synthesis	*PSD3*	Phosphatidylserine decarboxylase
*uqA9-10*	*qOCTO-A9-1* + *qICMO-A9-3*	*BnaA09g46210D*	*AT1G13560*	Eukaryotic phospholipid synthesis	*AAPT1*	Diacylglycerol cholinephosphotransferase
*uqC1-1*	*qICTO-C1-1*	*BnaC01g34540D*	*AT3G18000*	Eukaryotic phospholipid synthesis	*XPL1*	Phosphoethanolamine N-methyltransferase
*uqA1-2*	*qICMO-A1-1*	*BnaA01g21850D*	*AT1G58520*	GDSL	*RXW8*	
*uqA9-5*	*qICMO-A9-1* + *qRTO-A9-1*	*BnaA09g41930D*	*AT2G23540*	GDSL		
*uqA10-1*	*qOCTO-A10-1*	*BnaA10g09310D*	*AT5G55050*	GDSL		
*uqC1-1*	*qICTO-C1-1*	*BnaC01g37130D*	*AT3G14220*	GDSL		
*uqC3*	*qRMO-C3-1*	*BnaC03g44180D*	*AT2G04570*	GDSL		
*uqA8-1*	*qWSMO-A8-1* + *qICMO-A8-1* + *qOCMO-A8-1*	*BnaA08g08850D*	*AT4G18550*	Lipase		Acylhydrolase (DAD1-like)
*uqA9-3*	*qSCTO-A9-2*	*BnaA09g39950D*	*AT3G62590*	Lipase		Lipid acylhydrolase-like
*uqA9-5*	*qICMO-A9-1* + *qRTO-A9-1*	*BnaA09g42230D*	*AT2G22170*	Lipase		
*uqA9-6*	*qICMO-A9-2*	*BnaA09g44300D*	*AT1G19190*	Lipase		
*uqA9-7*	*qWSTO-A9-1* + *qWSMO-A9-2*	*BnaA09g44570D*	*AT1G18460*	Lipase		
*uqA9-12*	*qOCTO-A9-2* + *qWSTO-A9-2*	*BnaA09g51230D*	*AT1G02660*	Lipase		Lipid acylhydrolase-like
*uqA9-12*	*qOCTO-A9-2* + *qWSTO-A9-2*	*BnaA09g50430D*	*AT5G65158*	Lipase		
*uqC1-1*	*qICTO-C1-1*	*BnaC01g37230D*	*AT3G14075*	Lipase		Lipid acylhydrolase-like
*uqA9-1*	*qRMO-A9-1*	*BnaA09g36000D*	*AT3G56600*	Lipid signaling		Phosphatidylinositol-4-kinase &gamma;
*uqA9-1*	*qRMO-A9-1*	*BnaA09g36300D*	*AT3G56960*	Lipid signaling	*PIP5K4*	Phosphatidylinositol-phosphate kinase type IB
*uqA9-2*	*qRMO-A9-2* + *qSCMO-A9-2*	*BnaA09g37990D*	*AT3G59770*	Lipid signaling	*SAC9*	Sac domain-containing phosphoinositide phosphatase
*uqA9-4*	*qSCMO-A9-3*	*BnaA09g40500D*	*AT2G26420*	Lipid signaling	*PIP5K3*	Phosphatidylinositol-phosphate kinase type IB
*uqA9-6*	*qICMO-A9-2*	*BnaA09g43530D*	*AT2G20900*	Lipid signaling	*ATDGK5*	Diacylglycerol kinase
*uqA9-7*	*qWSTO-A9-1* + *qWSMO-A9-2*	*BnaA09g44740D*	*AT1G76690*	Lipid signaling	*OPR2*	Oxo-Phytodienoic acid reductase
*uqA9-9*	*qRTO-A9-2*	*BnaA09g45010D*	*AT1G17420*	Lipid signaling	*LOX3*	Lipoxygenase
*uqA9-10*	*qOCTO-A9-1* + *qICMO-A9-3*	*BnaA09g48610D*	*AT1G08980*	Lipid signaling	*ATAMI1*	Fatty acid amide hydrolase
*uqA9-10*	*qOCTO-A9-1* + *qICMO-A9-3*	*BnaA09g47830D*	*AT1G10900*	Lipid signaling		Phosphatidylinositol-phosphate kinase type IB
*uqA10-2*	*qOCTO-A10-2*	*BnaA10g14700D*	*AT5G20840*	Lipid signaling		Sac domain-containing phosphoinositide phosphatase
*uqC1-1*	*qICTO-C1-1*	*BnaC01g37150D*	*AT3G14205*	Lipid signaling		Sac domain-containing phosphoinositide phosphatase
*uqC1-1*	*qICTO-C1-1*	*BnaC01g37100D*	*AT3G14270*	Lipid signaling	*FAB1B*	Phosphatidylinositol-phosphate kinase type III
*uqC3*	*qRMO-C3-1*	*BnaC03g45030D*	*AT2G06050*	Lipid signaling	*OPR3*	Oxo-Phytodienoic acid reductase
*uqA1-2*	*qICMO-A1-1*	*BnaA01g21980D*	*AT1G59820*	Miscellaneous: lipid related	*ALA3*	Translocase
*uqA9-7*	*qWSTO-A9-1* + *qWSMO-A9-2*	*BnaA09g44980D*	*AT1G17500*	Miscellaneous: lipid related		Translocase
*uqA9-10*	*qOCTO-A9-1* + *qICMO-A9-3*	*BnaA09g46440D*	*AT1G13210*	Miscellaneous: lipid related	*ACA.l*	Translocase
*uqA9-12*	*qOCTO-A9-2* + *qWSTO-A9-2*	*BnaA09g50730D*	*AT1G04010*	Miscellaneous: lipid related	*PSAT1*	Phospholipid:acyl acceptor Acyltransferase
*uqC1-1*	*qICTO-C1-1*	*BnaC01g33520D*	*AT4G17480*	Miscellaneous: lipid related		Thioesterase (PPT1-like)
*uqC3*	*qRMO-C3-1*	*BnaC03g43340D*	*AT3G23530*	Miscellaneous: lipid related		Cyclopropane fatty acid synthase
*uqA8-2*	*qOCMO-A8-2*	*BnaA08g12350D*	*AT4G31810*	Mitochondrial fatty acid and lipoic acid synthesis		Mitochondrial enoyl-CoA hydratase
*uqA9-6*	*qICMO-A9-2*	*BnaA09g43570D*	*AT2G20860*	Mitochondrial fatty acid and lipoic acid synthesis	*LIP1*	Lipoate synthase
*uqC3*	*qRMO-C3-1*	*BnaC03g44210D*	*AT2G04540*	Mitochondrial fatty acid and lipoic acid synthesis		Ketoacyl-ACP synthase
*uqA1-5*	*qICMO-A1-2*	*BnaA01g26210D*	*AT3G19260*	Sphingolipid synthesis	*LAG1 HOMOLOG 2*	Ceramide synthase
*uqA8-1*	*qICMO-A8-1* + *qOCMO-A8-1* + *qWSMO-A8-1*	*BnaA08g10210D*	*AT4G22330*	Sphingolipid synthesis	*ATCES1*	Ceramidase
*uqA9-2*	*qRMO-A9-2* + *qSCMO-A9-2*	*BnaA09g39290D*	*AT3G61580*	Sphingolipid synthesis		Sphingobase-D8 desaturase
*uqA9-6*	*qICMO-A9-2*	*BnaA09g43940D*	*AT2G19880*	Sphingolipid synthesis		Glucosylceramide synthase (UDP-glucose-dependent)
*uqA9-10*	*qOCTO-A9-1* + *qICMO-A9-3*	*BnaA09g46200D*	*AT1G13580*	Sphingolipid synthesis	*LAG13*	Ceramide synthase

In addition, 54 genes (62.8%) were identified as the underlying genes of unique QTLs having a specific effect on specific seed tissues (Table 3), which revealed the specific function of some genes participating in oil synthesis and accumulation in corresponding tissues. For instance, three oil body-related genes, *BnaA01g14480D*, *BnaA10g09480D* and *BnaC01g34070D* (homologous to *AtOLEO1*, *AtPXG2* and *AT3G18570*), were mapped to WSMO, OCTO and ICTO, respectively. Homologous genes of *AT2G22230*, *AtACP4* and *AtKCS4* involved in fatty acid synthesis and elongation located in QTL CIs were identified as underlying genes related to MO, and *BnaC01g33800D* and *BnaA10g09480D* (homologous to *AtLPAT5* and *ATPXG2* involved in TAG synthesis) were identified to affect ICTO and OCTO, respectively. In addition, 14 candidate genes were mapped to be specifically related to oil accumulation in R seeds, including the TF *BnaC03. ABI3* is involved in regulating the transition between embryo maturation and early seedling development. *BnaA09g40180D*, *BnaA09g39950D* and *BnaA09g40500D*, which are annotated as being involved in TAG degradation, lipase and lipid signaling, were identified to be specific for oil content in the SC of seeds.

## Discussion

### 3D phenotyping of oil content in different tissues of seeds provides a novel strategy for genetic dissection for seed oil content

Understanding the genetic basis of oil formation is necessary for increasing the oil content in rapeseed. Research has revealed that the distribution of lipid is far from uniform in different seed tissues [24]. Therefore, dissecting the genetic basis for oil content formation in different seed tissues has great practical significance for rapeseed breeding. Phenotyping is crucial to reveal natural genetic variation accompanied by genomics in crops, so progress in phenotyping technologies is required to accelerate genetic mapping and gene discovery [53]. Most of the current methods for the determination of oil content in rapeseed are either destructive [9–11, 45, 54] or difficult to realize quantitative visualization of lipids in seeds [12–14, 40–42]. MRI has been successfully used to evaluate the relative contribution of different tissues to the overall visualization and quantity of lipid accumulated in various seeds, including *B. napus* seeds [24, 26, 55], which provides possibilities for QTL mapping based on the oil content of different seed tissues. In this study, by combining lipid MRI and 3D quantitative analysis, we reveal the lipid distribution, which overcomes many drawbacks existing in oil content determination, such as the influence of the gap between the slices on lipid quantitation and the destruction of seeds in the follow-up experiment [11, 24, 26, 45], and precision is compromised when the seed shape is not the same as the model seed for imaging [14, 18]. Here, our study provides a novel method for the acquisition of phenotypic data of oil content for QTL mapping and the dissection of the genetic basis for oil content in different seed tissues.

Conventional breeding for high oil content has always relied on improvements in the capacity of the organs appropriate for lipid storage [56–58]. Lipid maps showed that the deposit level of oil was significantly different in different tissues, even within a tissue, which indicated that the breeding ideas can be further expanded. Thus, the currently rendered lipid mapping of the different tissues of seeds allows the identification of more precise ‘topographical targets’ for breeding in the future. Since the cotyledons made the largest contribution to the oil content, the seed oil content could be increased more if the oil content in the dark areas of OC and IC was improved in *B. napus* (Fig. 1a–c). In addition, the proportion of oil content in SC and R was 18.58% and 10.52% of the volume in the whole seed, respectively; however, the proportion of oil content in SC and R was 2.54% and 5.78% in N53-2 with high seed oil content (51.74%) (Additional file 1: Fig. S1). The results suggest that the oil content could increase by further increasing the oil content of cotyledons and radicles or selecting seeds with thin seed coats and larger cotyledons (especially outer cotyledons) to reach a level equivalent to that achieved in cotyledons, as suggested by Borisjuk et al. [26]. Even though the dark areas of the seeds were exits of the vascular system for directing the flow of water [59], after a preliminary calculation, we deduced that the oil content of rapeseed varieties with 60% might be expected to reach up to 75% when the oil content in the middle dark zone of the cotyledon reached the average level of lipids in the surrounding area of the cotyledon.

### New perspective for QTL mapping for oil content in *B. napus* seeds

In recent years, numerous QTLs for oil content have been identified in *B. napus* [32–37]. These QTLs explained PV ranging from 1.2% to approximately 20% [36, 60, 61], while in previous reports, all QTLs for seed oil content were based on the oil content of the whole seeds. To our knowledge, this genetic dissection of seed oil content based on QTL mapping in the different tissues of seeds is the first reported. More QTLs for oil content were identified when the whole seed was subdivided into four tissues (Table 1). Four QTLs (*uqA8-1*, *uqA9-7*, *uqA9-10* and *uqA9-12*) were identified corresponding to QTLs that were identified based on the same population and genetic map [62]. These QTLs were related to MO or TO of WS, OC or IC, in which *uqA9-7* and *uqA9-12* were the two unique QTLs that control the only two WSTO-QTLs (*qWSTO-A9-1* and *qWSTO-A9-2*), respectively. In addition, the QTL genomic regions of two unique QTLs, *uqA9-1* (*qRMO-A9-1*) and *uqA9-2* (*qRMO-A9-2* and *qSCMO-A9-2*), and four unique QTLs, *uqA9-6* (*qICMO-A9-2)*, *uqA9-7* (*qWSMO-A9-2* and *qWSTO-A9-1*), *uqA9-7* (*qOCMO-A9-2*) and *uqA9-7* (*qRTO-A9-2*), were mapped to be identical to *TN-qOC-A9-3* and *TN-qOC-A9-4*, respectively [28]. Another two QTLs (*qOCMO-A8-2* and *qOCTO-A10-2*, with small effects) were also identified in a previous study [62]. Especially important, 14 tissue-specific QTLs were identified, including some with major effects. In addition, *uqA9-6,* with up to 12.63% of PV, was a specific QTL for the average oil content of IC and colocalized with *TN-qOC-A9-4*. Moreover, the BSA results further confirmed the common QTLs identified in this study and Chao et al. [62]. These results indicated that QTL mapping based on MRI was accurate and credible, and it could provide new insights into variation loci of oil content derived from the contribution of which tissues when more detailed information was provided. Here, 11 QTLs were thought to be novel and were not identified in all previous studies, seven of which were tissue-specific QTLs. Among them, *uqA9-11* was considered to be a main QTL controlling ICMO, OCMO, RTO and WSMO. In our study, it was revealed that accumulating favorable alleles for TO in different tissues, especially the OC, may be an effective way to increase the WSTO. Adding favorable alleles for RTO and OCTO could significantly increase the WSTO (Fig. 6e). The cumulative effects of QTLs of different tissues thus revealed that seed oil content is partly controlled by tissue-specific quantitative genes with mainly additive effects. Our study indicates that tissue-specific selection for seed oil content should be effective in breeding for oil content enhancement, which further emphasizes the urgent need to develop tissue-specific molecular markers for molecular-assisted selection breeding of high oil content varieties.

### Analysis of candidate genes demonstrates the application value of 3D phenotyping strategies

Transcriptome analysis for each seed tissue was performed to offer further support for candidate identification in combination with tissue-specific QTL mapping. In terms of TAG biosynthesis-related genes, IC, OC and R showed the same gene expression characteristics, FA synthesis was more active at 24 DAP, and TAG synthesis was more active at 33 DAF (Fig. 7 and Additional file 1: Fig. S8), which reflected lipid synthesis regularity in the developmental process of seeds. Some important candidate genes related to lipid synthesis and metabolism in the CIs of QTLs were identified in the QTL interval. *CAC2*, *KCS17* and *FAE1*, which are directly involved in fatty acid (FA) synthesis and elongation, were identified in the CI of pleiotropic QTLs that control OC and IC. The *BnaA09. CAC2*, which is located in both OC-QTL and IC OC-QTL, showed higher expression levels in OC and IC of N53-2 at 24 and 33 DAF, which will promote the flow of precursors to FA synthesis in OC and IC.

Ten candidate genes involved in phospholipase, aliphatic suberin synthesis, lipase, sphingolipid synthesis, fatty acid elongation and cuticular wax synthesis, such as *BnaA08. FAE1*, *BnaA08. KCS17*, *BnaA08. CER4* and *BnaA08. LPT1*, were mapped in CI of *uqA8-1,* which controls ICMO, OCMO and WSMO. More erucic acid was detected in the seeds of N53-2 than Ken-C8 [63], although *BnaA08. FAE1*, which has been reported to determine the erucic acid content [64], had not been detected to be differentially expressed in OC and IC between the two parents, and we are more likely to believe that it might be only one causal gene, *BnaA08. FAE1* controlled ICMO, OCMO and WSMO, which needs to be further verified by fine mapping. Interestingly, three genes, *BnaA09g38540D*, *BnaA09g38630D* and *BnaA09g39290D,* in the overlapping CI of *qRMO-A9-2* and *qSCMO-A9-2* were annotated to be involved in cuticular wax synthesis, sulfolipid synthesis and sphingolipid synthesis, respectively, instead of TAG metabolism, which would provide new clues to further explore oil content variation in R and SC. In addition, the genes involved in TAG degradation, lipase and lipid signaling were identified to be specific for oil content in SC, which would provide some evidence for the high accumulation of specific fatty acids in the SC, such as C16:1 [26]. In the annotation of DEGs, SC showed greater differences than IC, OC and SC between the two parents, and important TFs and key structural genes, such as *WRI1*, *LEC1/2*, *CAC2* and *KASII,* involved in FA synthesis were differentially expressed, which suggested that FA biosynthesis was significantly different in SC between the high oil content N53-2 and the low oil content Ken-C8. However, the data from 3D phenotyping analysis showed that no oil content difference in SC was found between the two parents (Additional file 2: Table S1). What is the reason for this? We also found that flavonoid and phenylpropanoid biosynthesis pathways were significantly enriched for SC-specific DEGs, which might indicate that the synthesized fatty acids are degraded for the synthesis of flavonoids simultaneously. This discovery provided new insights for candidate identification of oil content QTLs in SC.

In addition, we focused on the candidate genes within CIs of tissue-specific QTLs of IC and OC. *BnaA01g14480D*, *BnaA10g09480D* and *BnaC01g34070D* were mapped in the CIs of *qWSMO-A1-1*, *qOCTO-A10-1* and *qICTO-C1-1*. They were homologous to three oil-body genes, *AtOLEO1*, *AtPXG2* and *AT3G18570*, and might be related to the specific growth and building of oil bodies in OC and IC. A large number of candidate genes involved in FA and TAG synthesis were mapped to underlying oil variation of OC or IC, for example, *AT2G22230*, *AtACP4*, *LPAT5* and *ATPXG2*, which demonstrates the utility of 3D phenotyping strategies for genetic dissection of the mechanism for oil formation of different tissues of seeds in *B. napus*.

## Conclusions

The phenotype based on whole seeds was unable to sufficiently reflect the complex genetic characteristics of seed oil content. In this study, the 3D distribution of lipids was measured for *B. napus* seeds by MRI and 3D quantitative analysis, and ten novel oil content-related traits were obtained by subdividing the seeds into different tissues. Based on a high-density genetic linkage map, 35 QTLs were identified for 4 tissues of OC, IC, R and SC with up to 13.76% of the phenotypic variation, and 14 tissue-specific QTLs were the first reported, including 7 novel QTLs. Haplotype analysis showed that the favorable alleles for different seed tissues exhibited cumulative effects on oil content. Tissue-specific transcriptomes revealed that more active energy and pyruvate metabolism influenced carbon flow in IC, OC and R than in SC at the early and middle seed development stages, thus affecting the distribution difference in oil content. Combining tissue-specific QTL mapping and transcriptomics, 86 important candidate genes associated with lipid metabolism were identified that underlie 19 unique QTLs, including the fatty acid synthesis rate-limiting enzyme-related gene *CAC2*, in QTLs for OC and IC. The present study provides further insight into the genetic basis for improving seed oil content at the tissue-specific level.

## Materials and methods

### Plant materials

Three *B. napus* lines from the KN DH population [49] with different oil content (51.74%, 46.90% and 40.10%) were selected for magnetic resonance imaging (MRI) analysis in different seed tissues. The KN DH population was derived from microspore culture of F1 plants after hybridization between ‘N53-2’ with high oil content (approximately 50%) and male ‘KenC-8’ with low oil content (approximately 40%) [49]. In addition, a random subset of 200 lines from the KN DH population was used for oil content analysis in different seed tissues using MRI. All materials were planted in a field located in Dali of Shaanxi Province (winter rapeseed planting area, E109.93°, N34.80°), China (sown in September 2014 and harvested in May of the following year). The field experiment was performed following standard breeding field protocols as previously described [49], and the seeds of all lines were collected after maturation.

### Noninvasive 3D phenotypic analysis and total and mean oil content determination in different seed tissues

MRI experiments were performed as previously described [24] on a vertical 11.75 T wide bore (inner diameter 89 mm) Bruker Avance III spectrometer (Bruker BioSpin GmbH, http://www.bruker-biospin.com/, Rheinstetten, Germany) at 500.13 MHz and equipped with an actively shielded Micro 2.5 gradient system (inner diameter of 40 mm and maximum strength 1 T/m). For MRI analysis, the mature dry seeds were placed into a home-built 5-mm ID solenoid coil, and a global T1 measurement of the lipid signal was conducted to determine the minimum repetition time (TR) necessary to avoid T1 correction during the quantification process. Considering the gap between planes, it could not sufficiently reveal the lipid distribution and lipid quantitative information when using the 2D sequence in MRI [24, 26]. Here, a 3D-FISP sequence with a CHESS suppression module on the water resonance was applied to acquire high-resolution lipid images of the seeds. Eventually, a voxel resolution of up to 30 μm isotropic in the seeds could be achieved (experimental time 10 h 40 min).

To accelerate 3D phenotyping, a special scaffold was produced to hold 12 rapeseed seeds at the same time for imaging. After obtaining data, in-house software was used for data reconstruction. The visualization and manual segmentation for modeling were performed with the 3D visualization software package Amira 5.4.0 (Visage Imaging GmbH, http://www.visageimaging.com/) based on standard manipulation procedures [24]. After segmentation, the average signal and total signal intensity in each tissue was obtained (Additional file 3: Video SI). After imaging, the seeds (approximately 4 mg) were immersed in 1.5 ml of 2.5% H_2_SO_4_ methanol solution with 0.01% butylated hydroxytoluene (BHT), 0.4 ml methylbenzene, and 200 μl of 2 mg/ml C17:0 as an internal standard following nitrogen filling. The mixture was incubated at 90 °C for 1 h. Then, 1.8 ml of deionized water and 1 ml of hexane were added to the mixture, and the supernatant was transferred into vials for gas chromatography (GC) analysis. GC was performed as previously described [65], and the actual oil content was calculated by the flame ionization detector response of sample components relative to 17:0 internal standards.

The measured MRI signals of the seeds were plotted vs. the known total lipid content of the seeds through Microsoft Office Excel 2010. The fitted calibration curve was used to quantify the oil content in different tissues of the rapeseed seeds, providing quantitative information on lipid distribution.

### Trait evaluation and statistical analysis, QTL mapping and meta-analysis

According to the method established above, the total lipid signal intensity, volume and mean signal intensity for different seed tissues were obtained for each line from a DH population (*n* = 200) after segmentation was conducted with Amira 5.4.0. After calibration, the total oil content (TO) and the mean oil content (MO) of each seed were determined. In total, 10 oil content-related characteristics in different tissues of seeds in the KN DH population were obtained, which included the whole seed oil content (WSTO), outer cotyledon total oil content (OCTO), inner cotyledon total oil content (ICTO), radicle total oil content (RTO), seed coat total oil content (SCTO), whole seed mean oil content (WSMO), outer cotyledon mean oil content (OCMO), inner cotyledon mean oil content (ICMO), radicle mean oil content (RMO) and seed coat mean oil content (SCMO). The statistical analysis of the phenotypic data of 10 traits was conducted using Microsoft Office Excel 2010 and SPSS 20.0 software.

A high-density genetic linkage map containing 101 non-SNP (SSR, STS) and 3106 SNP-bin markers constructed as previously described [62] was employed for QTL mapping. QTL identification was implemented using the composite interval mapping (CIM) model in Windows QTL Cartographer V2.5 [49]. Parameters were set for the CIM model as described by Chao et al. [62]. The LOD threshold was determined for significant QTLs by a 1,000-permutation test based on a 5% experimental error rate. Significant QTLs identified in each trait were termed ‘identified QTLs’. The method for assigning nomenclature to identified QTLs was described in Wang et al. [49]. The identified QTLs were named by combining the trait and the chromosome number, e.g., *qOCTO-A10-1*, representing the first QTL identified for OCTO on chromosome A10. QTLs for different traits with overlapping CIs were integrated into unique QTLs using the QTL meta-analysis method in the BioMercator V4.2 program [62].

### Identification of genomic regions and candidate genes underlying QTLs for oil content

The collinearity analysis of the linkage map and the *B. napus* “Darmor-bzh” reference genome [66] was conducted based on SNP probes as described previously [67]. Genomic regions corresponding to the CI of QTLs were defined through both closely linked SNPs within QTL CIs and colinearity between the linkage map and the reference genome. If genes that fell within the QTL genomic region were annotated to be related to lipid metabolism, they were regarded as candidate genes. The genomic regions of QTLs from other populations, DY [36], RNSL [36], and TN [28], were obtained from electronic PCR (e-PCR) [68] for comparison with the QTLs identified in the present study, which was performed with the primer sequences of the molecular markers flanking the QTL confidence intervals using the genomic sequences of ‘Darmor-bzh’ as templates. The orthologs of candidate genes within QTL regions and their annotations were obtained by BLASTn based on the *A. thaliana* database (TAIR: http://www.arabidopsis.org/). In addition, BSA analysis based on extremely high and low oil content lines in the KN population was from a previous study [62].

### RNA extraction, transcriptome sequencing and DEG identification

IC, OC, R and SC tissues of seeds N53-2 and Ken-C8 at 24 and 33 days after flowering (DAF) were separated using a stereomicroscope and then flash-frozen in liquid nitrogen. Three biological replicates were collected, and total RNA was extracted from each seed tissue using an RNAprep pure plant kit (DP432, http://www.tiangen.com/). Extracted RNA samples were sent Novogene Corporation (Beijing, China) for library construction and transcriptome sequencing on an Illumina HiSeq 2500 platform. Approximately, 6 Gb of Illumina cleaned reads were collected for each tissue per sample type. Low-quality reads, connectors, and barcode sequences were eliminated using Trimmomatic-0.39. All downstream analyses were based on clean data with high quality. Then, the clean data were aligned to the reference genome of ‘Darmor-bzh’ [66] using HISAT (v2.2.1, https://www.nature.com/articles/nmeth.3317). Gene expression levels were quantified as count number and FPKM using the programs featurecounts and cuffquant, respectively [69]. Differentially expressed genes (DEGs) were identified using the R package DEseq 2 based on the criteria of false discovery rate (FDR, Benjamini–Hochberg multiple test correction) < 0.01 and absolute fold change > 2 [70].

## Supplementary Information

**Additional file 1: Fig. S1. **Total and mean oil content in different tissues of rapeseed seeds with different oil content.andrepresent the total and mean oil content in different tissues of rapeseed seeds, respectively. **Fig. S2. **Quantitative imaging of lipids in different tissues of seeds in the KN DH population based on three-dimensional reconstruction. 1, 2, 3, 4, 5 and 6 represent the whole seed, seed coat, inner cotyledon, outer cotyledon, radicle and seed section, respectively. **Fig. S3.** Pearson correlation coefficients for trait pairs affecting the oil content of rapeseed seeds in the KN DH population. **Fig. S4. **Distribution of identified QTLs for oil content in different tissues of seeds in the A1, A8, A9, A10, C1, C3 and C9 linkage groups. WOC refers to identified QTLs for relative oil content detected by near-infrared spectroscopy. **Fig. S5. **The correlations among all 48 samples in different tissues at the two seed sampling stages of Ken-C8 and N53-2. **Fig. S6. **KEGG enrichment of the tissue-specific DEGsin the four tissues at 24 and 33 DAP. **Fig. S7. **The expression characteristics of genes involved in fatty acid synthesis, TAG synthesisand β-oxidationin four tissues of Ken-C8 and N53-2 at 24 and 33 DAF. **Fig. S8. **The expression characteristics of genes involved in fatty acid synthesis, TAG synthesisand β-oxidationin four tissues of Ken-C8 and N53-2 at 24 and 33 DAF.

**Additional file 2: Table S1.** Mean values and phenotypic variation for oil content in different tissues of seeds in the KN DH population. **Table S2.** Pearson correlation coefficients for trait pairs affecting the oil content of seeds in the KN DH population. **Table S3.** The allele type of each DH line in 12 accessed TO-QTLs. **Table S4.** Characteristics of sequencing data for RAN-Seq.

**Additional file 3: Video S1. **3D lipid phenotyping in different tissues of seeds in the mapping population.

## Data Availability

All data generated or analyzed during this study are included in this published article and its supplementary information files.
