# Supplementary material for: New insight into the genetic basis of oil content based on noninvasive three-dimensional phenotyping and tissue-specific transcriptome in Brassica napus
Source: Biotechnol Biofuels Bioprod. 2023 May 23;16:88. doi: 10.1186/s13068-023-02324-0 (PMC10207806; doi:10.1186/s13068-023-02324-0)
Supplement: Supplementary file 1 — Additional file 1: Fig. S1. Total and mean oil content in different tissues of rapeseed seeds with different oil content.andrepresent the total and mean oil content in different tissues of rapeseed seeds, respectively. Fig. S2. Quantitative imaging of lipids in different tissues of seeds in the KN DH population based on three-dimensional reconstruction. 1, 2, 3, 4, 5 and 6 represent the whole seed, seed coat, inner cotyledon, outer cotyledon, radicle and seed section, respectively. Fig. S3. Pearson correlation coefficients for trait pairs affecting the oil content of rapeseed seeds in the KN DH population. Fig. S4. Distribution of identified QTLs for oil content in different tissues of seeds in the A1, A8, A9, A10, C1, C3 and C9 linkage groups. WOC refers to identified QTLs for relative oil content detected by near-infrared spectroscopy. Fig. S5. The correlations among all 48 samples in different tissues at the two seed sampling stages of Ken-C8 and N53-2. Fig. S6. KEGG enrichment of the tissue-specific DEGsin the four tissues at 24 and 33 DAP. Fig. S7. The expression characteristics of genes involved in fatty acid synthesis, TAG synthesisand β-oxidationin four tissues of Ken-C8 and N53-2 at 24 and 33 DAF. Fig. S8. The expression characteristics of genes involved in fatty acid synthesis, TAG synthesisand β-oxidationin four tissues of Ken-C8 and N53-2 at 24 and 33 DAF. [file 13068_2023_2324_MOESM1_ESM.docx]

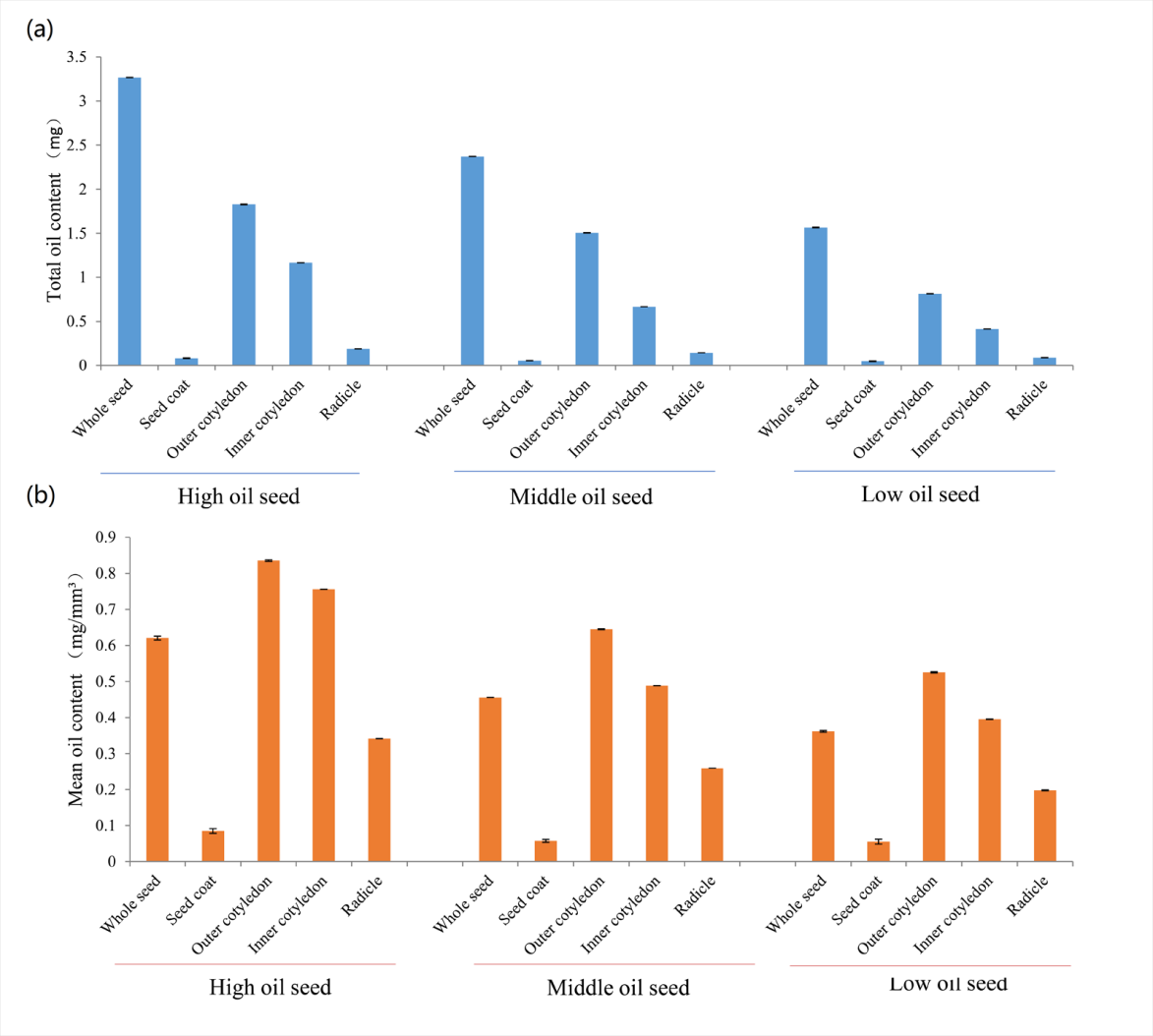


**Supplementary Figures 1.** Total and mean oil content in different tissues of rapeseed seeds with different oil content. (a) and (b) represent the total and mean oil content in different tissues rapeseed seeds, respectively.


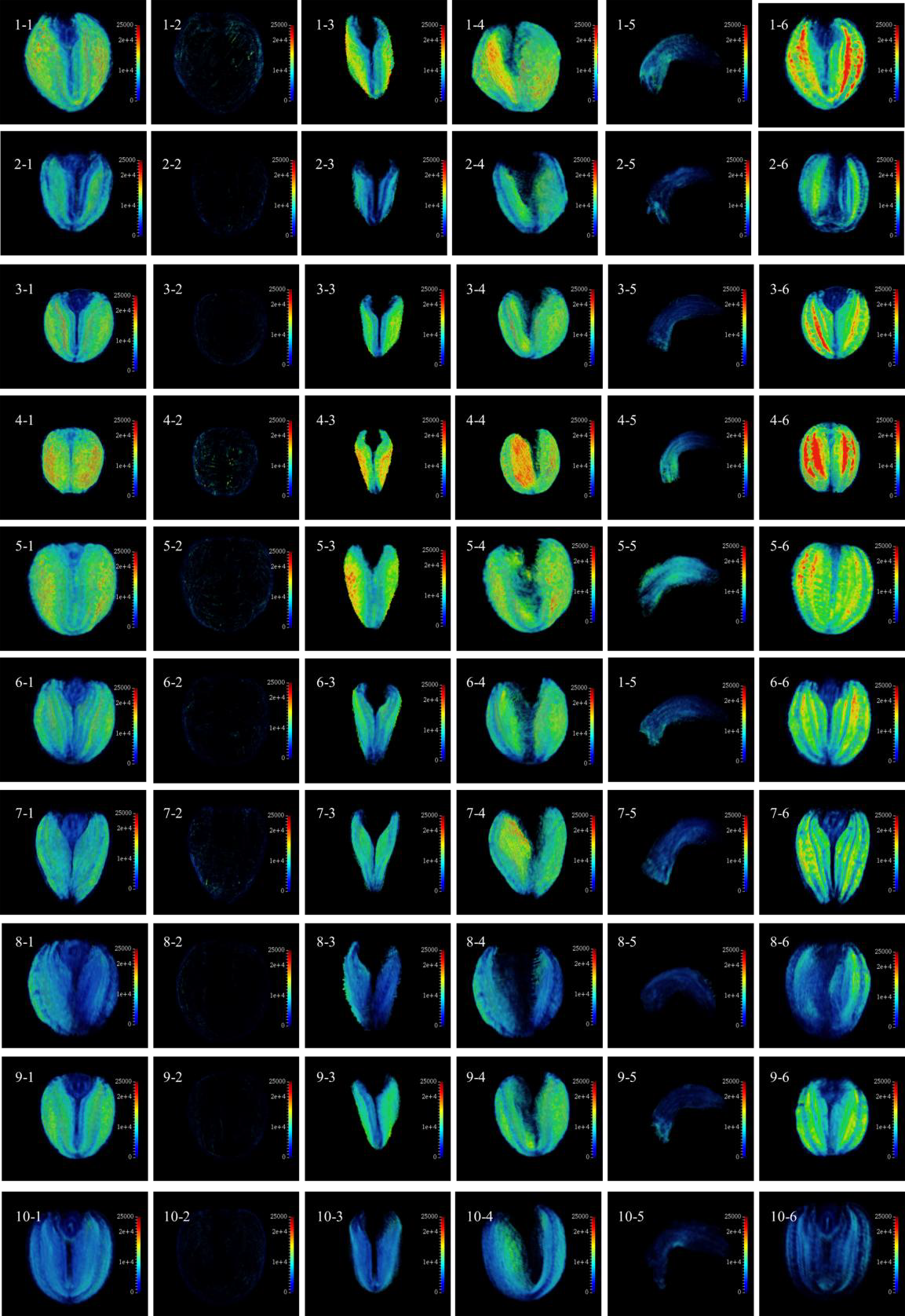


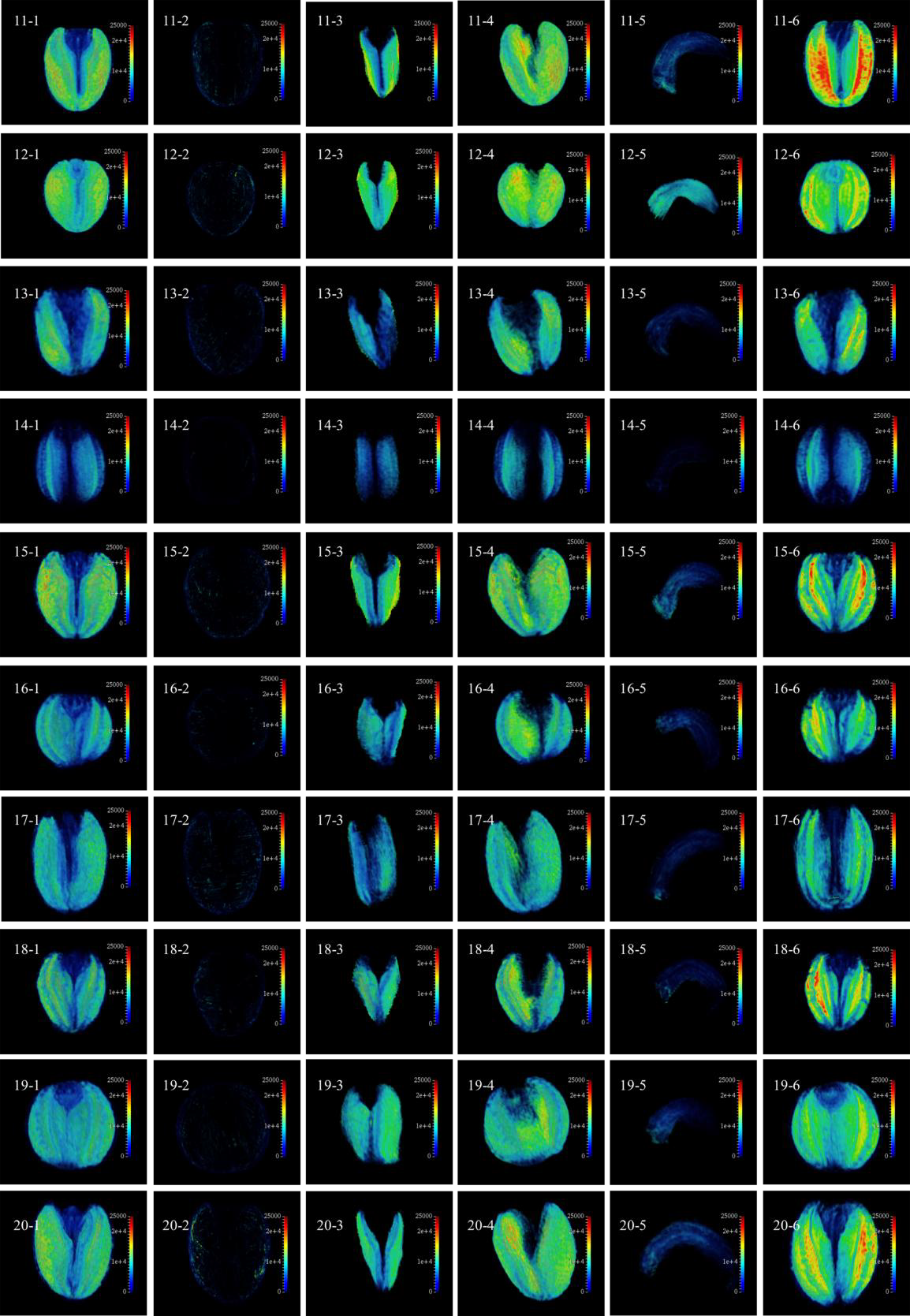


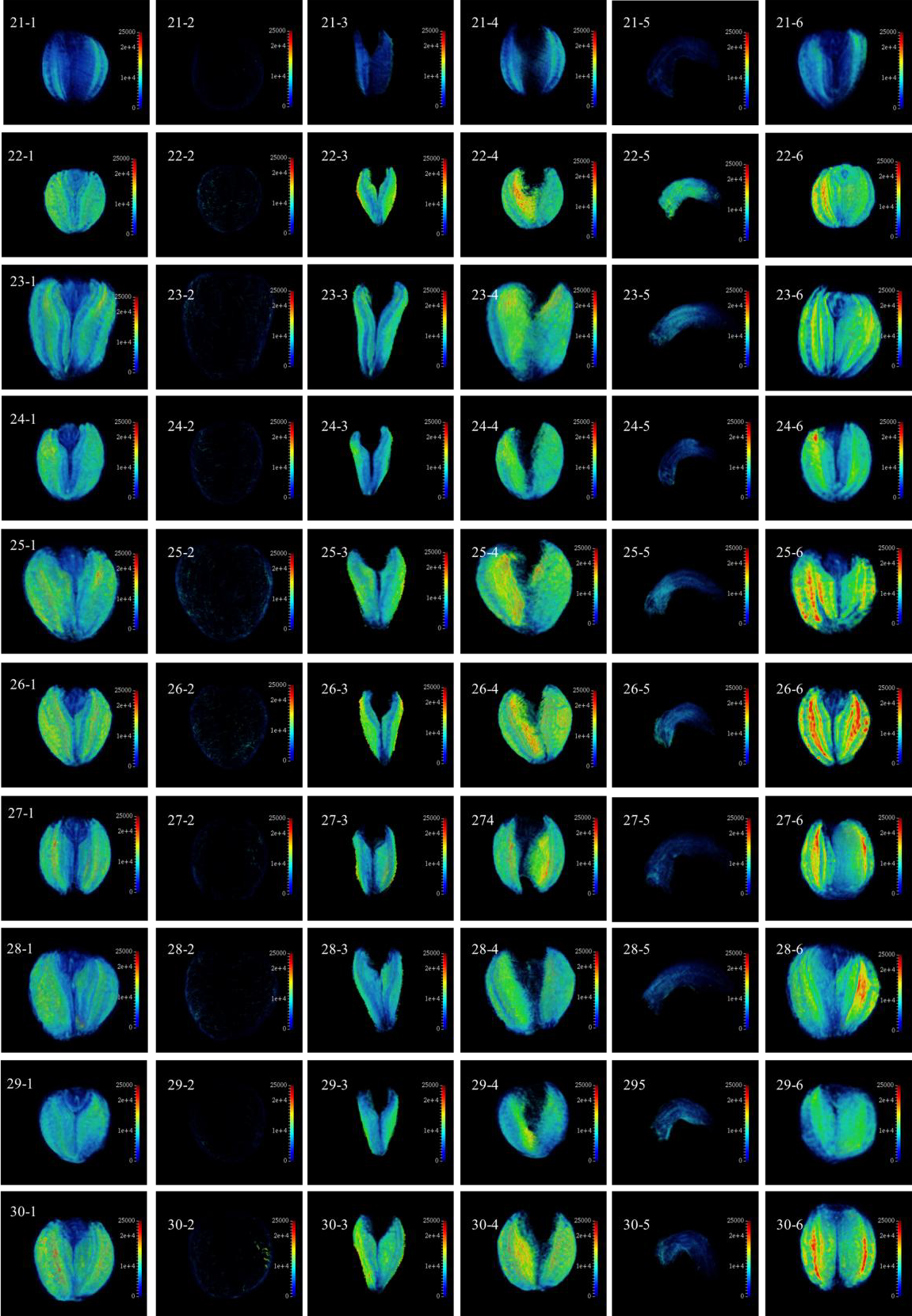


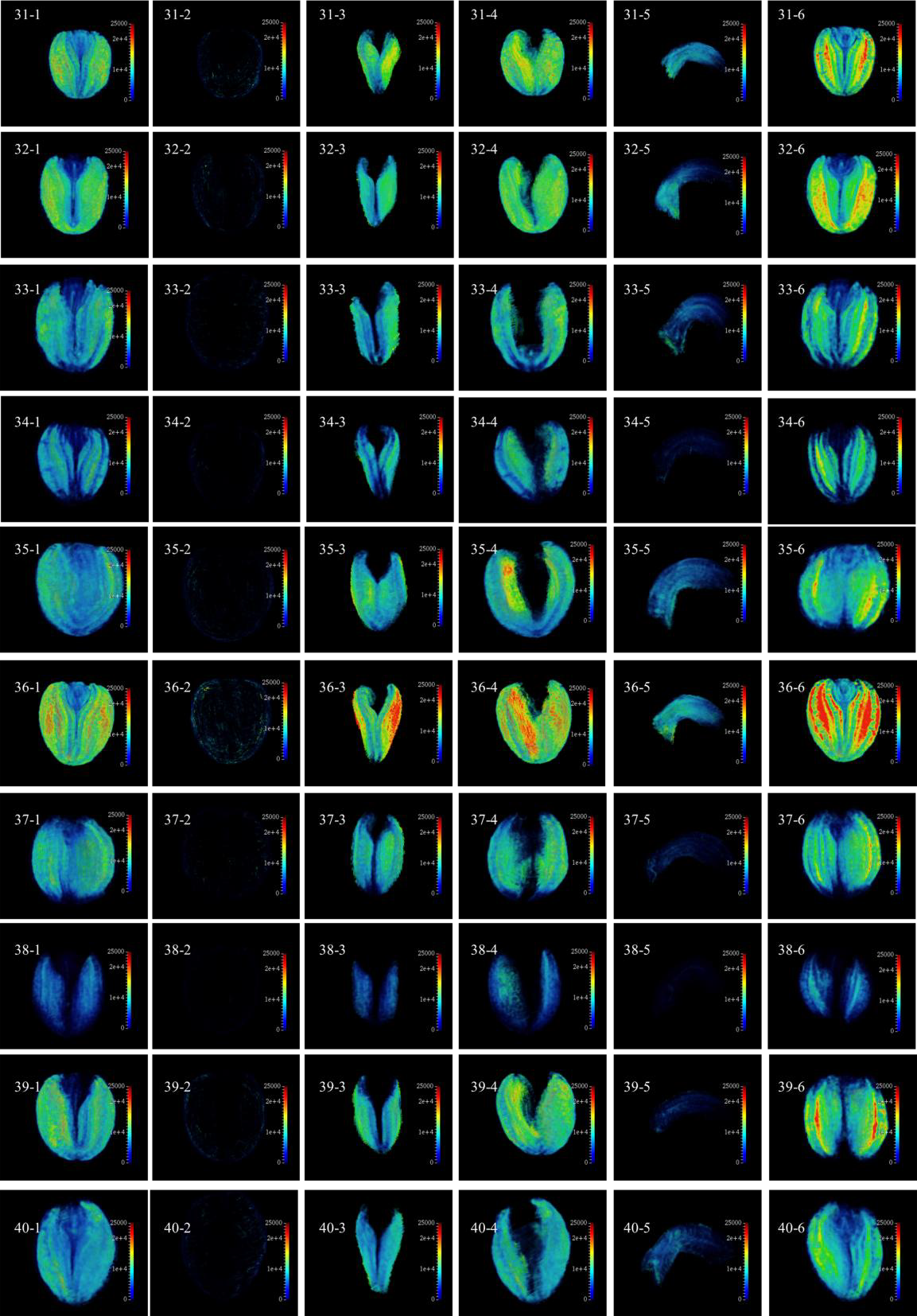


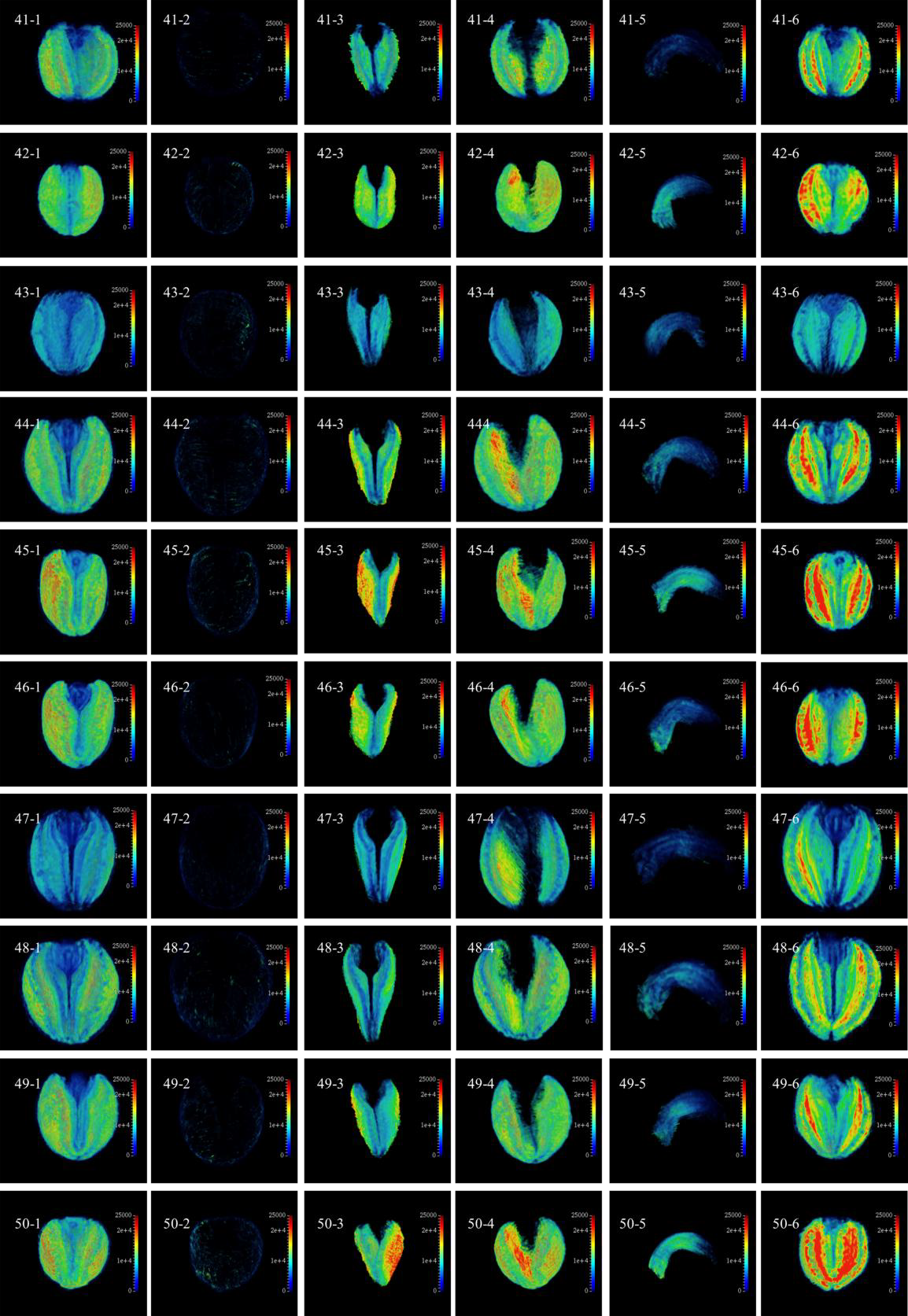


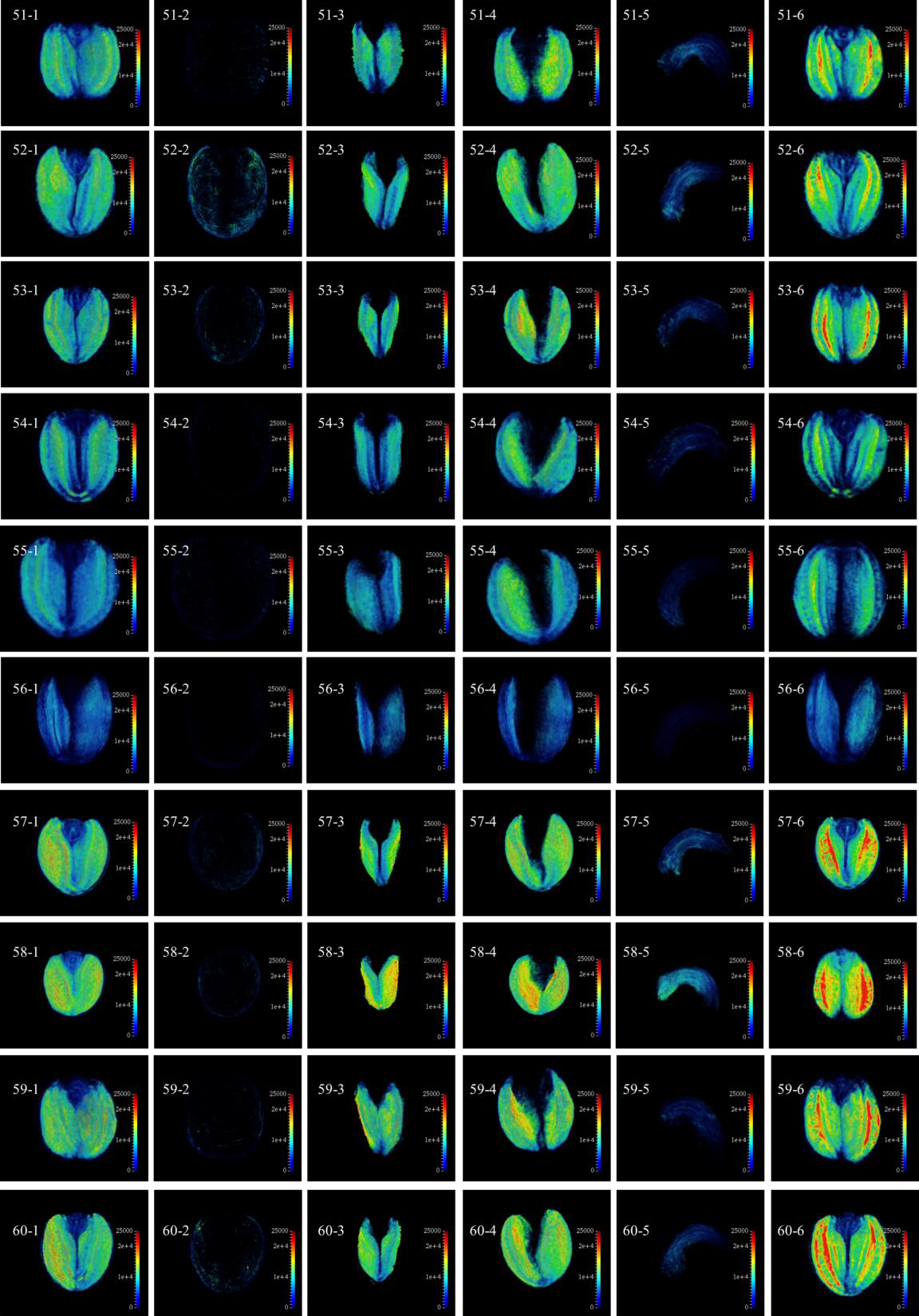


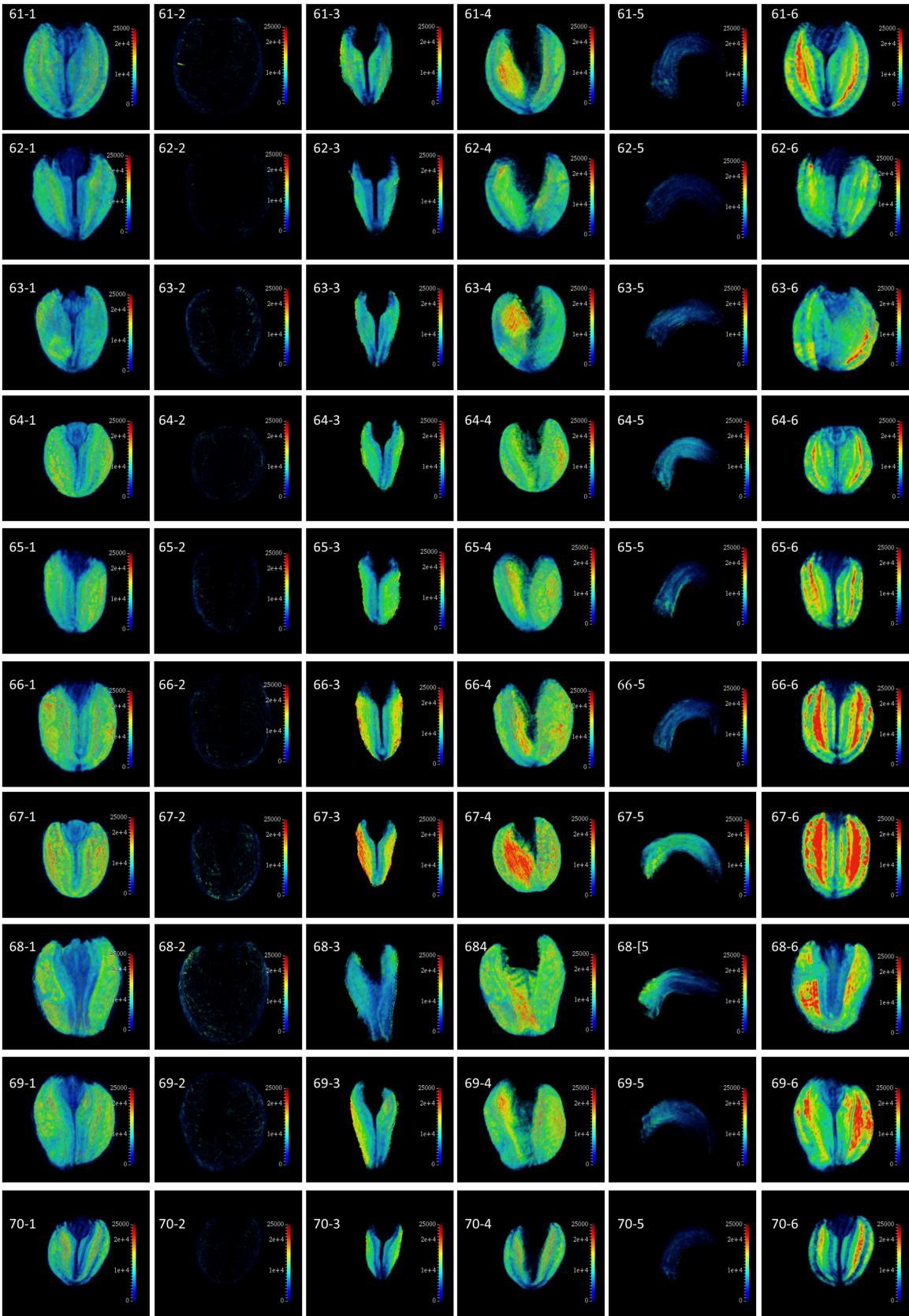


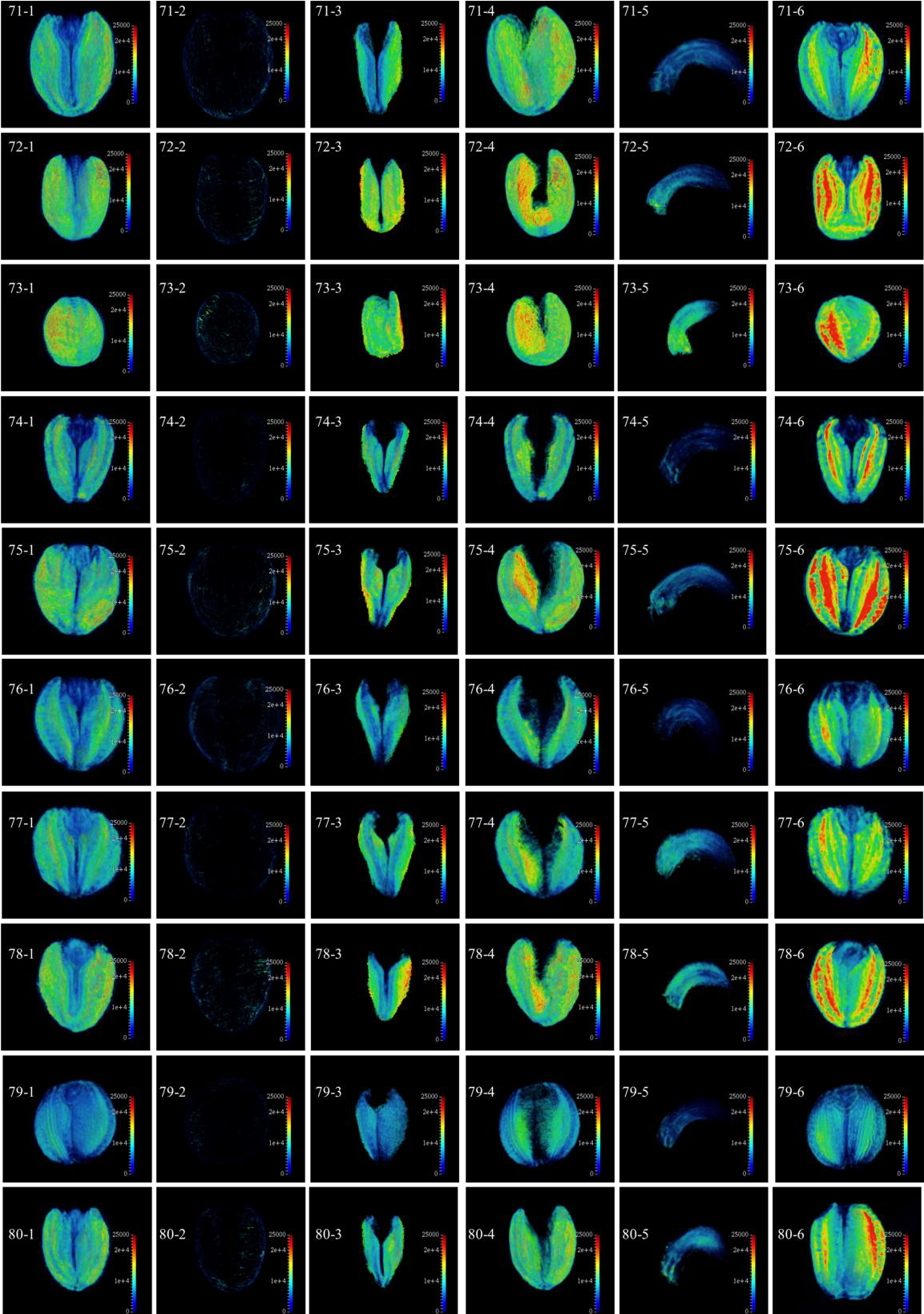


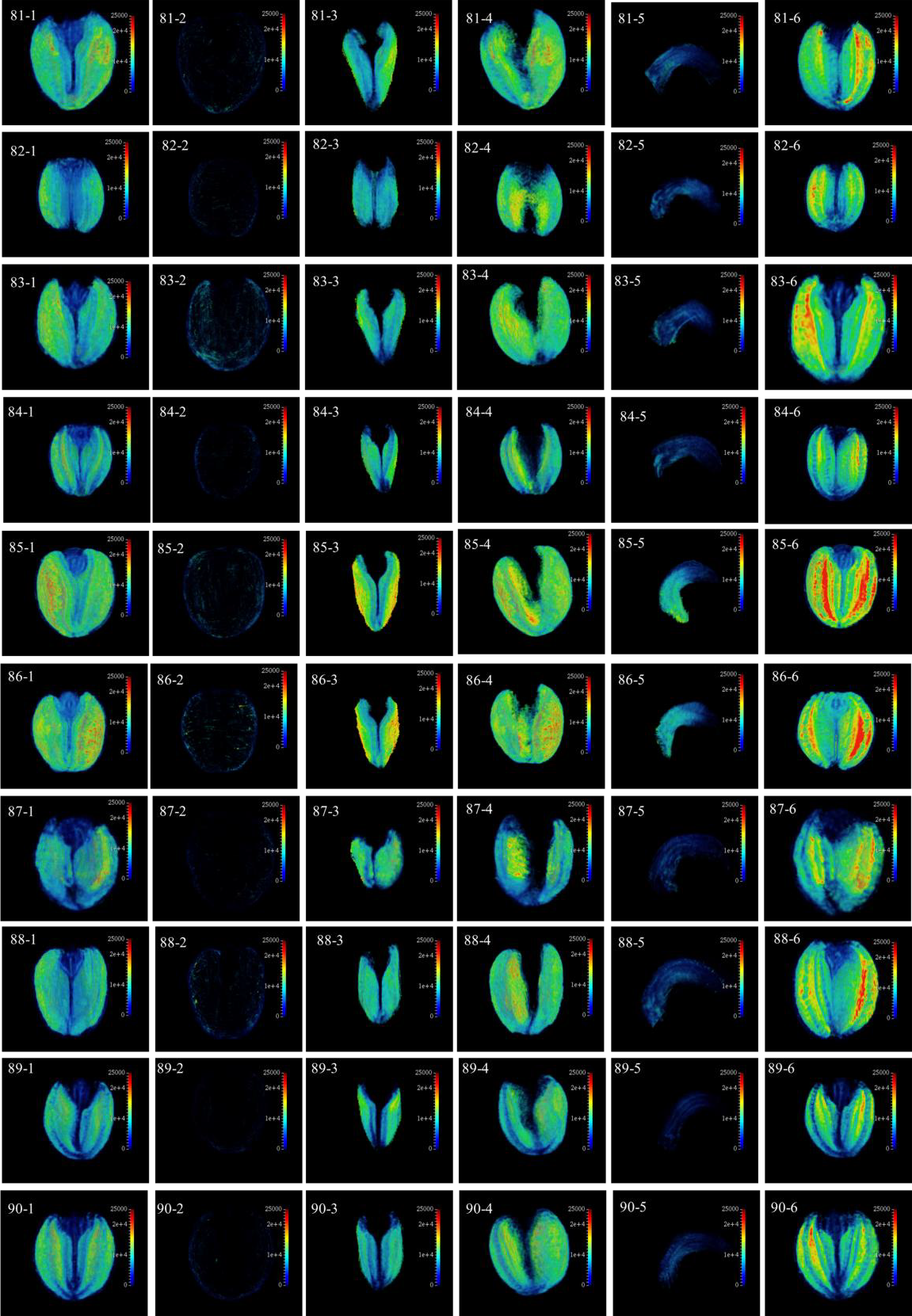


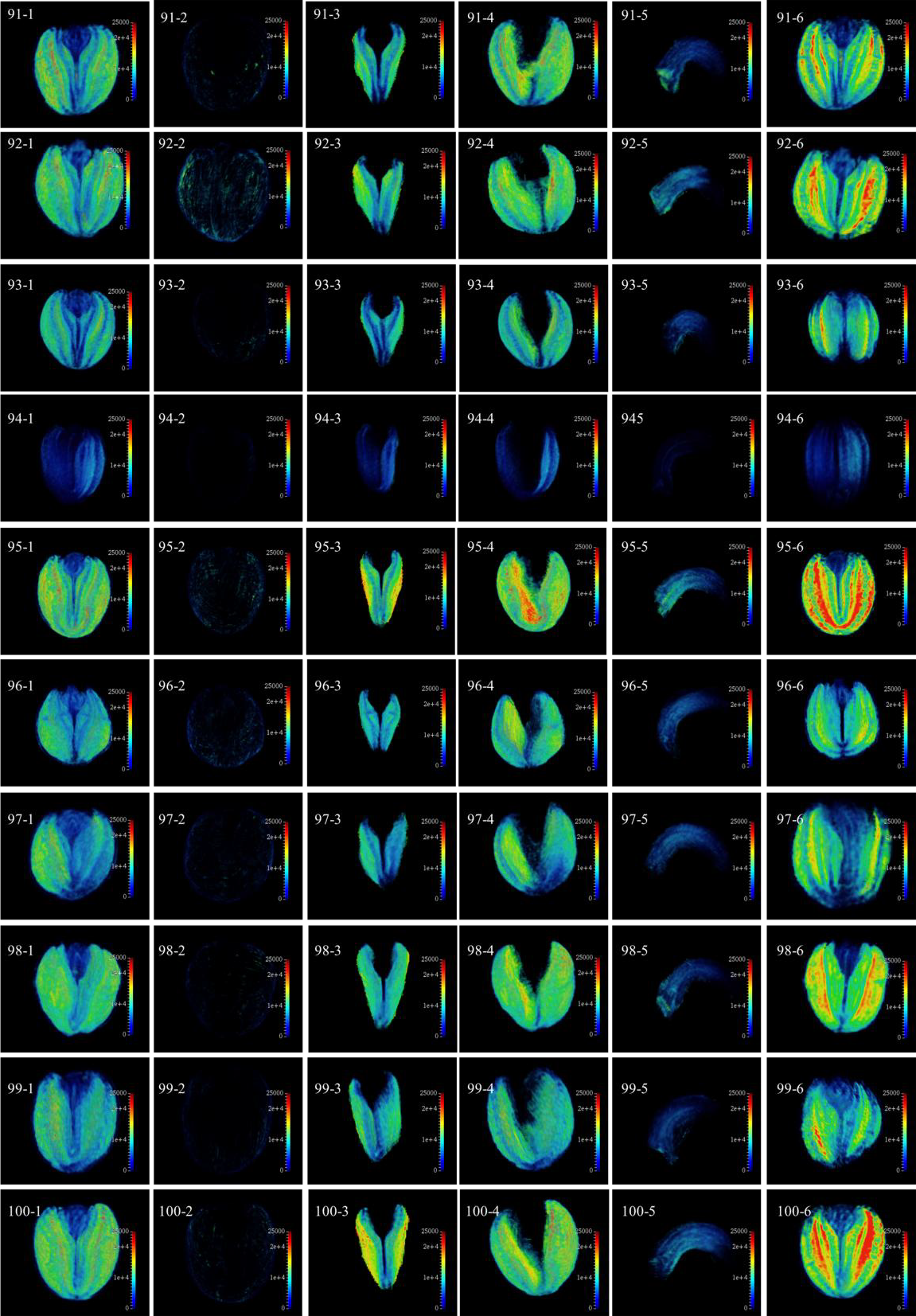


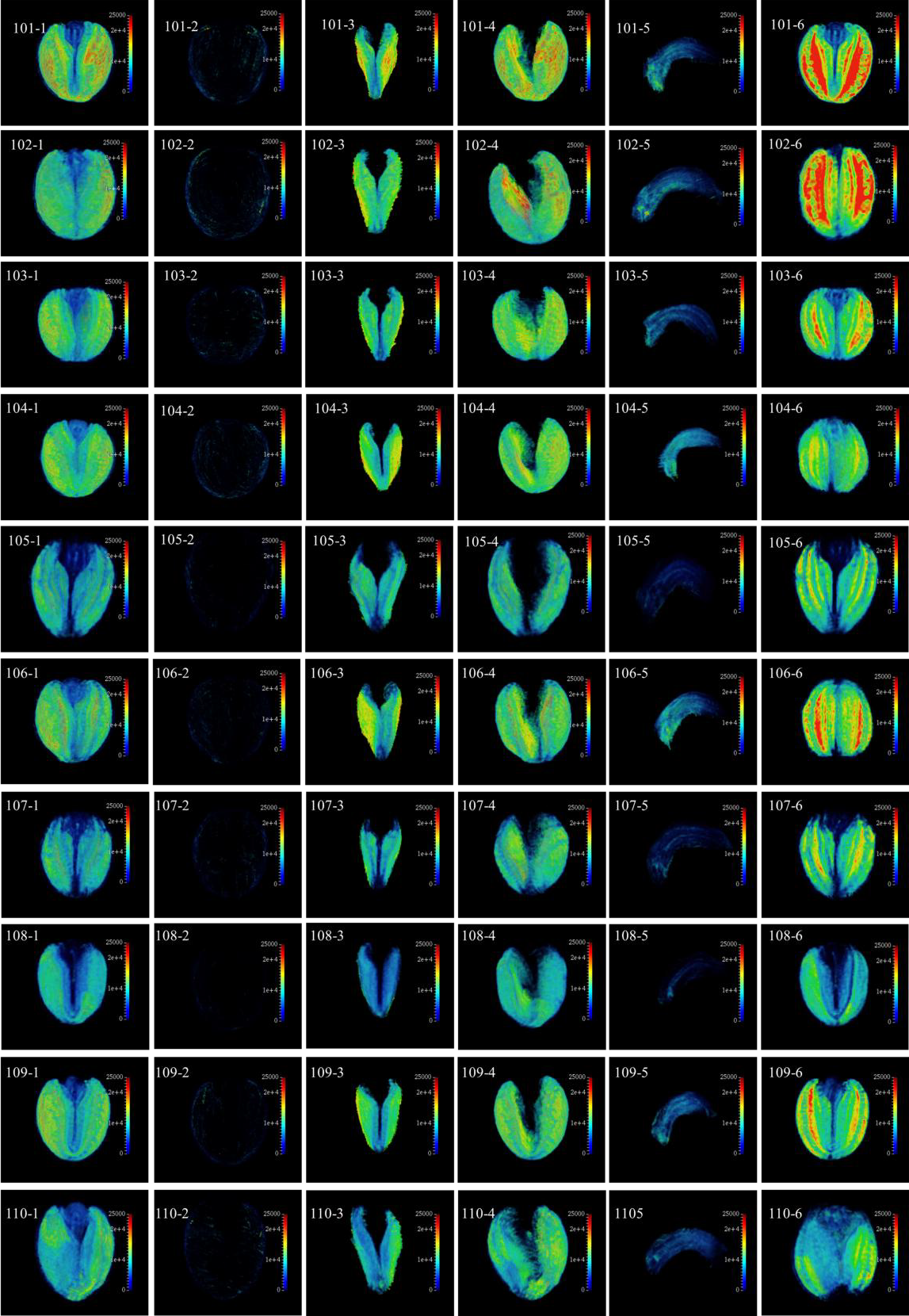


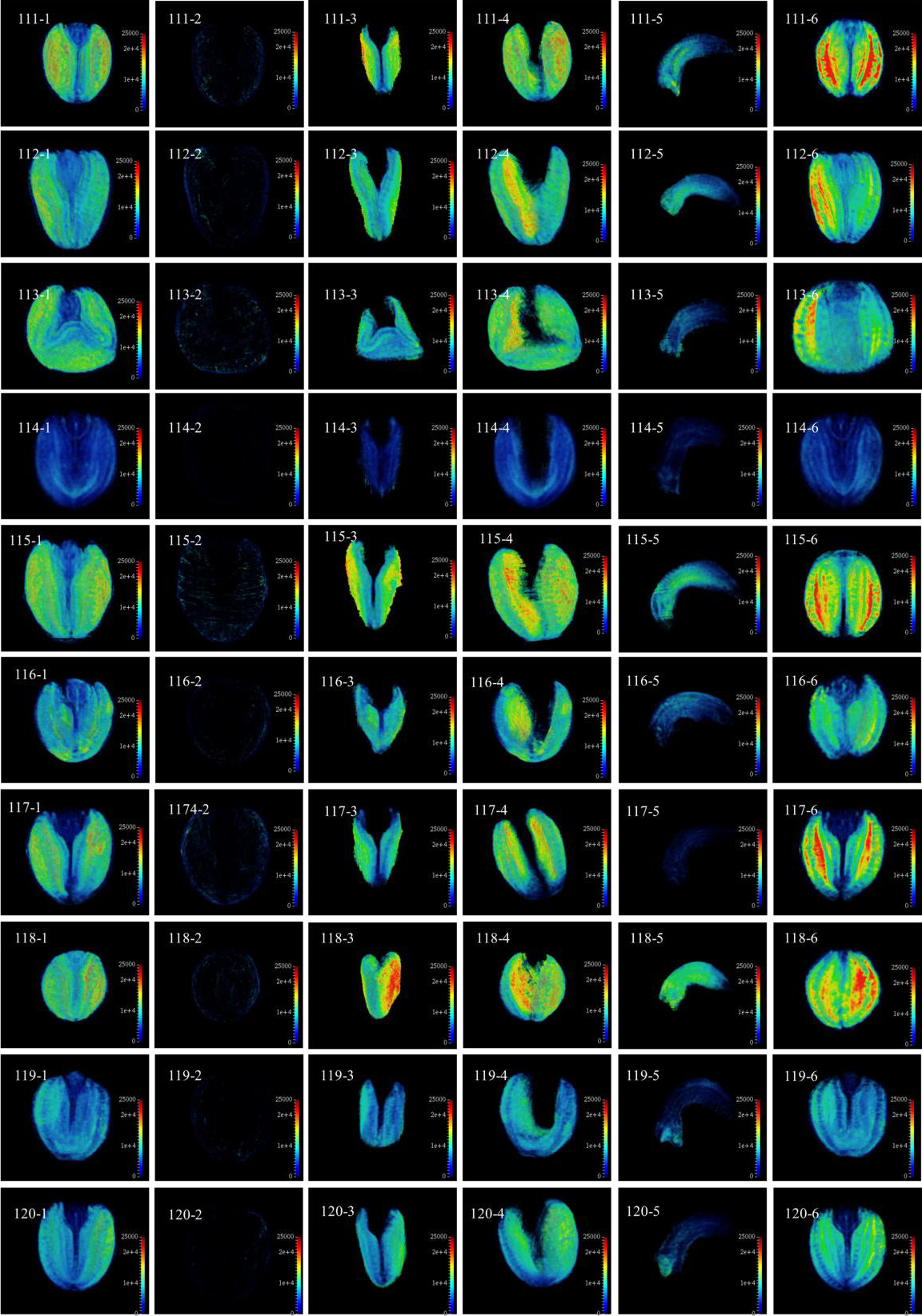


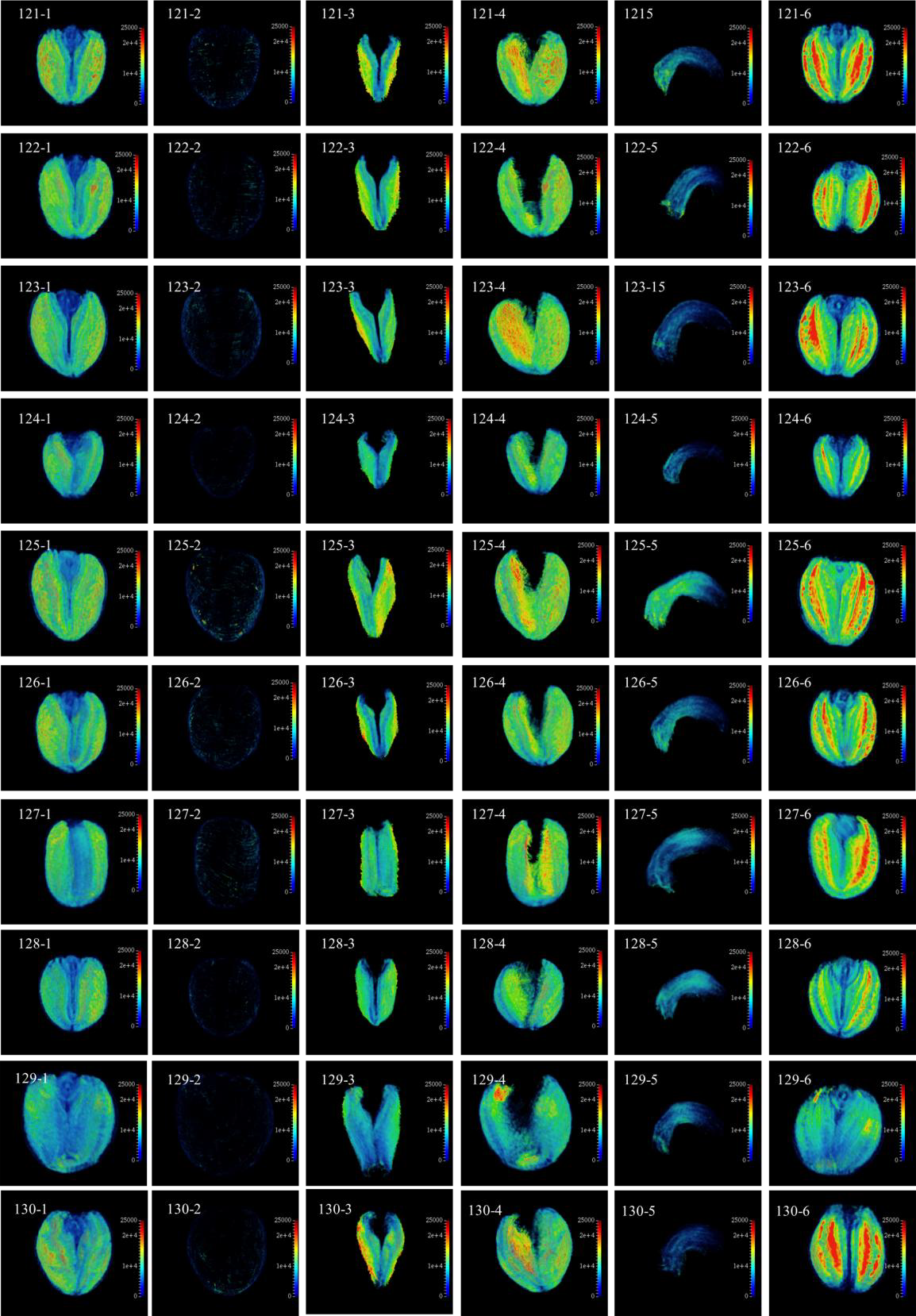


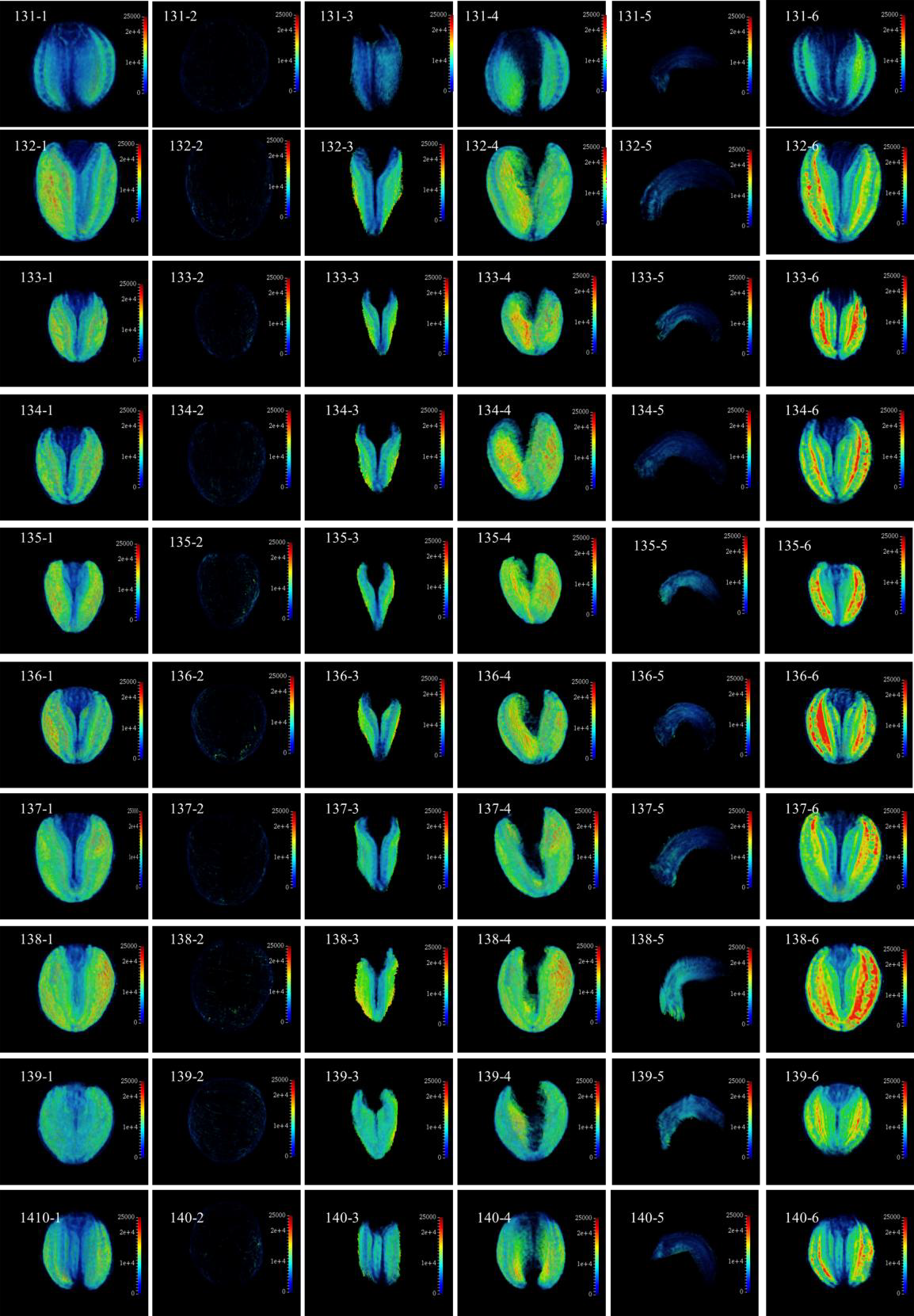


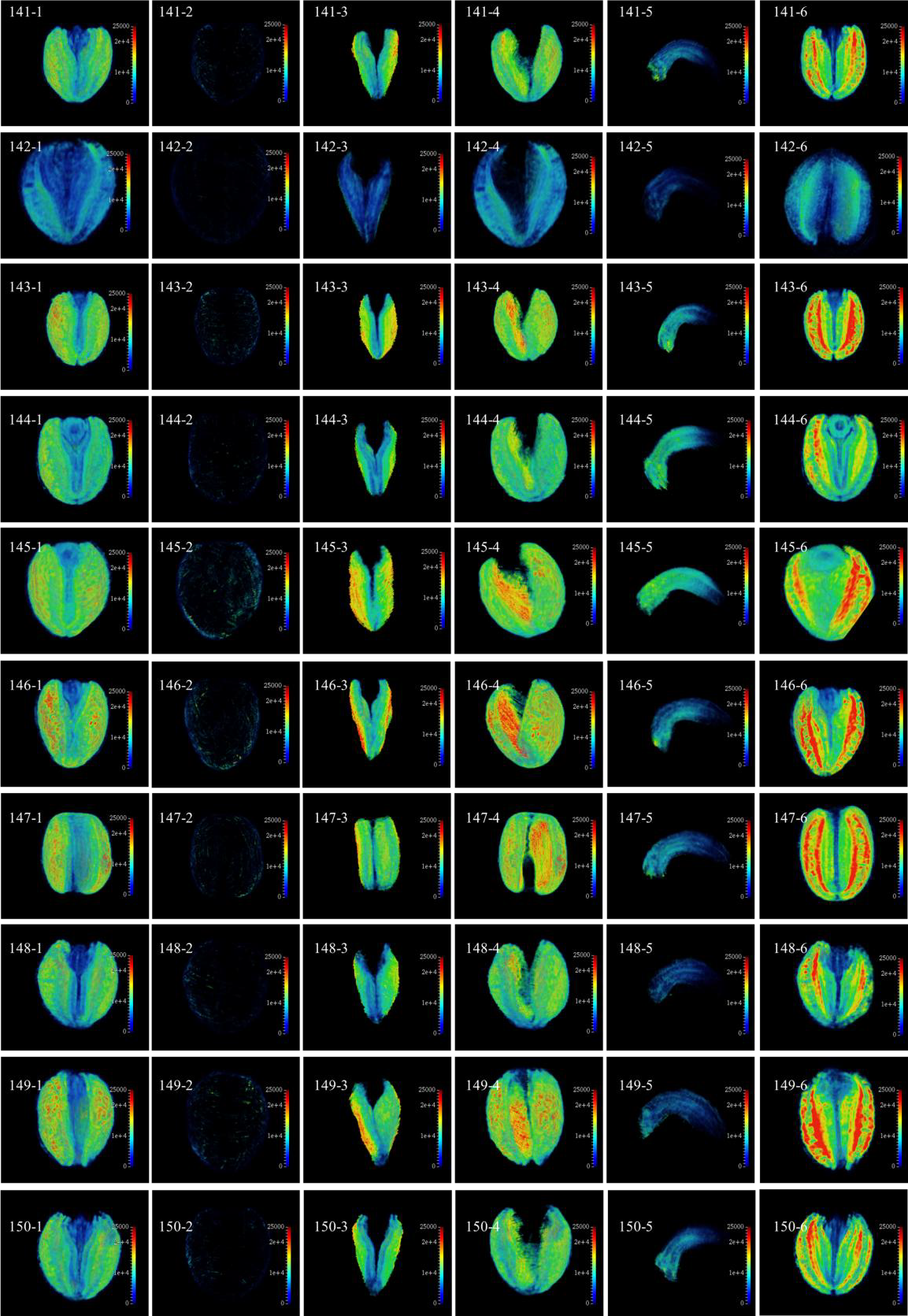


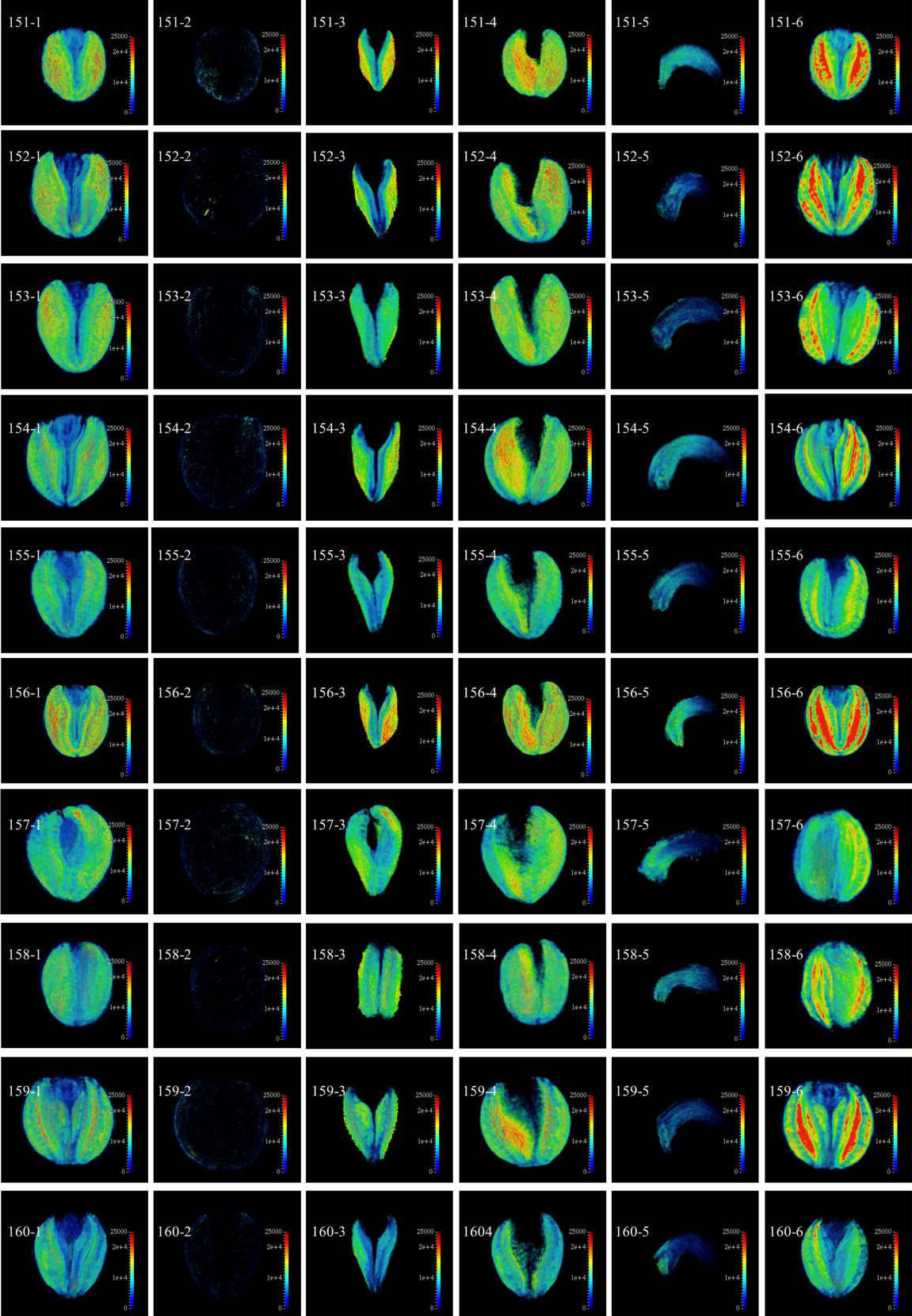


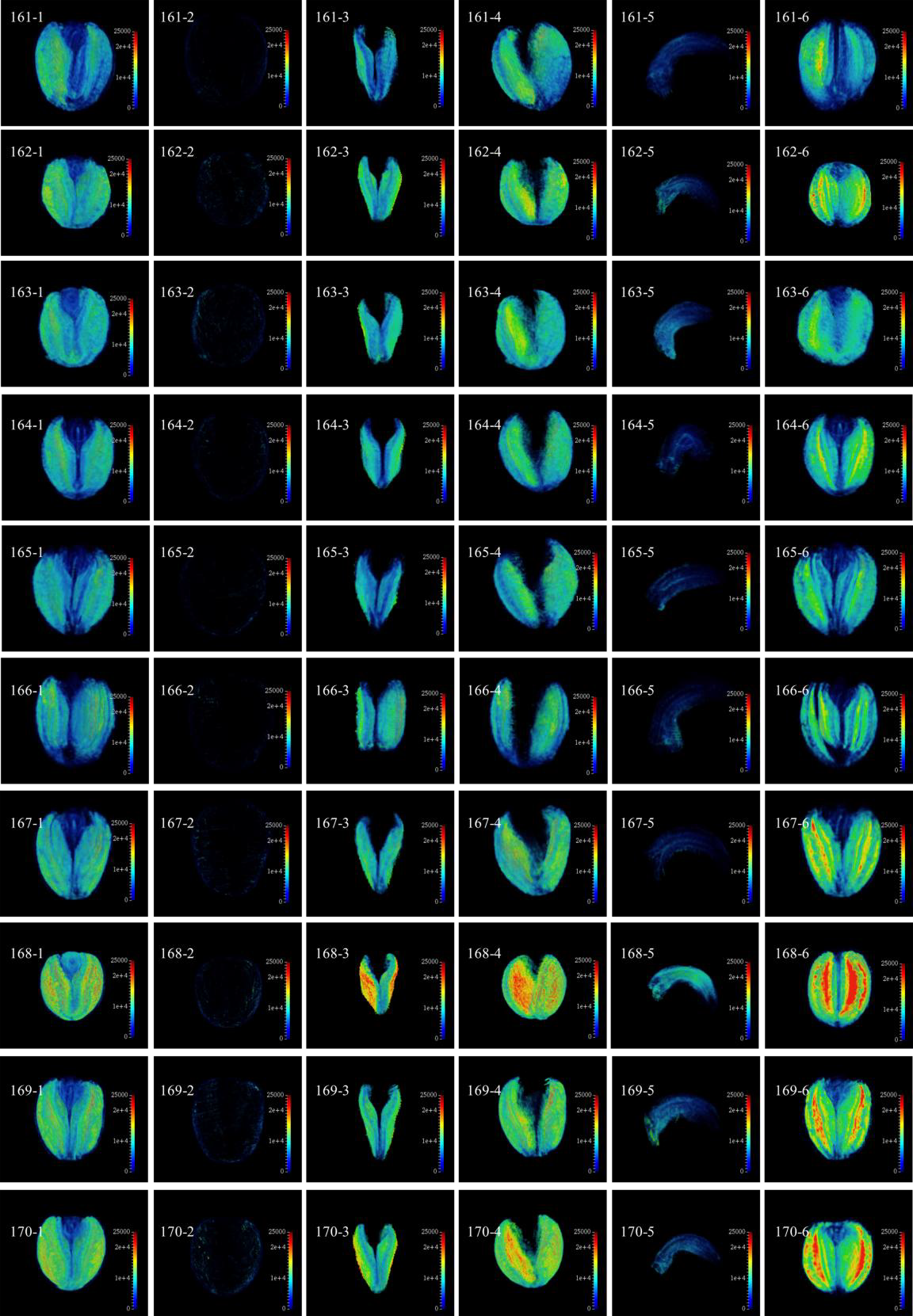


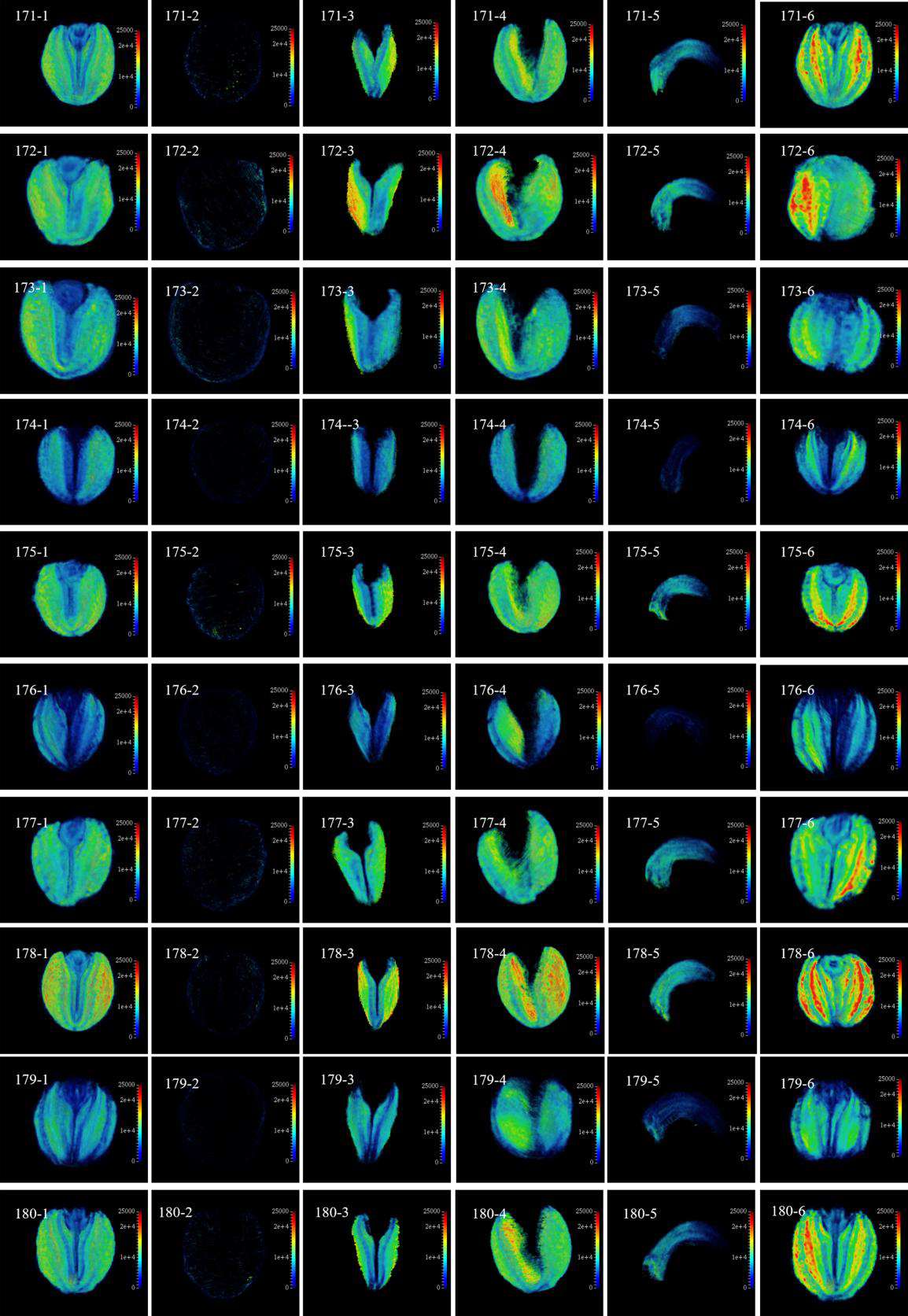


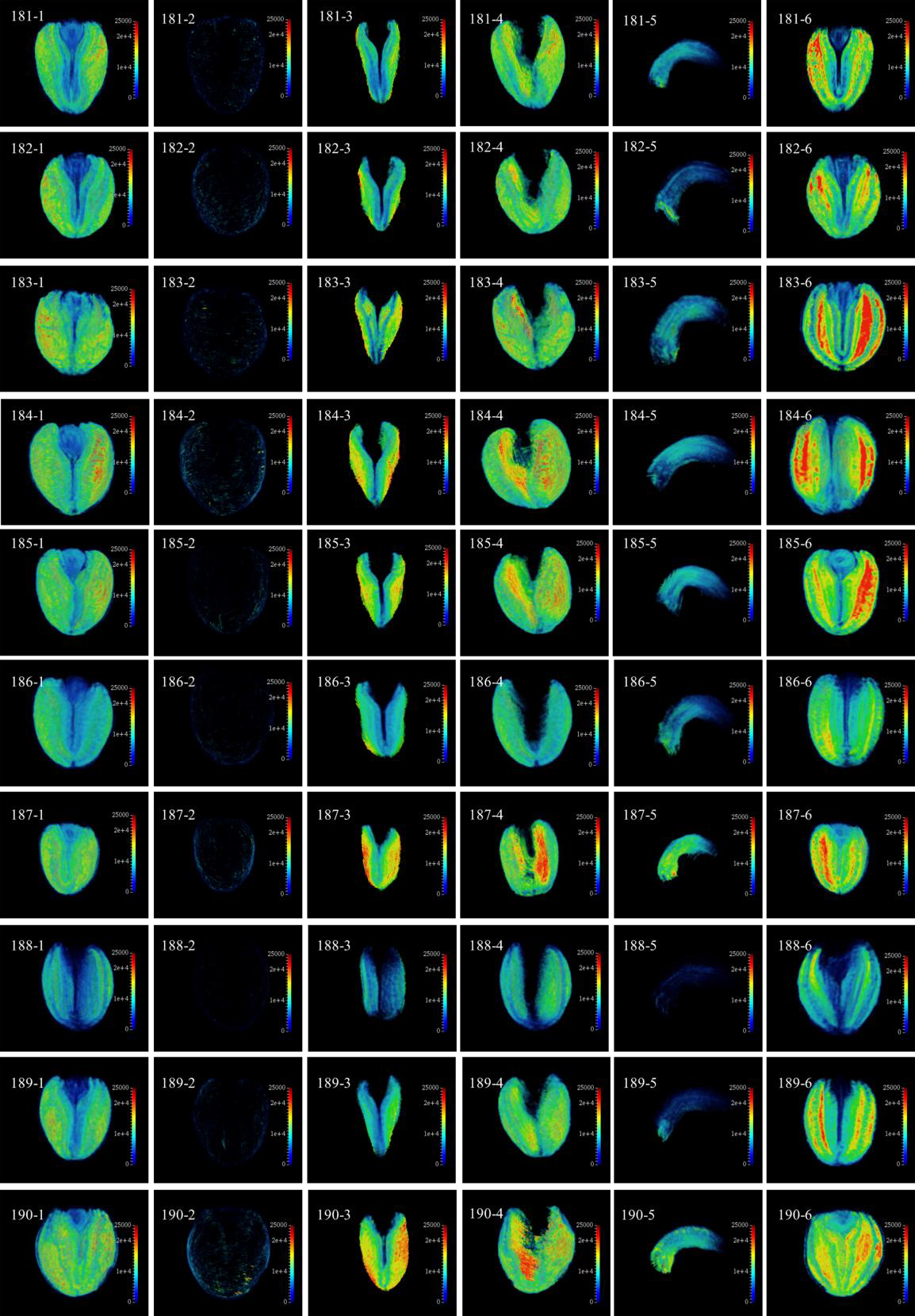


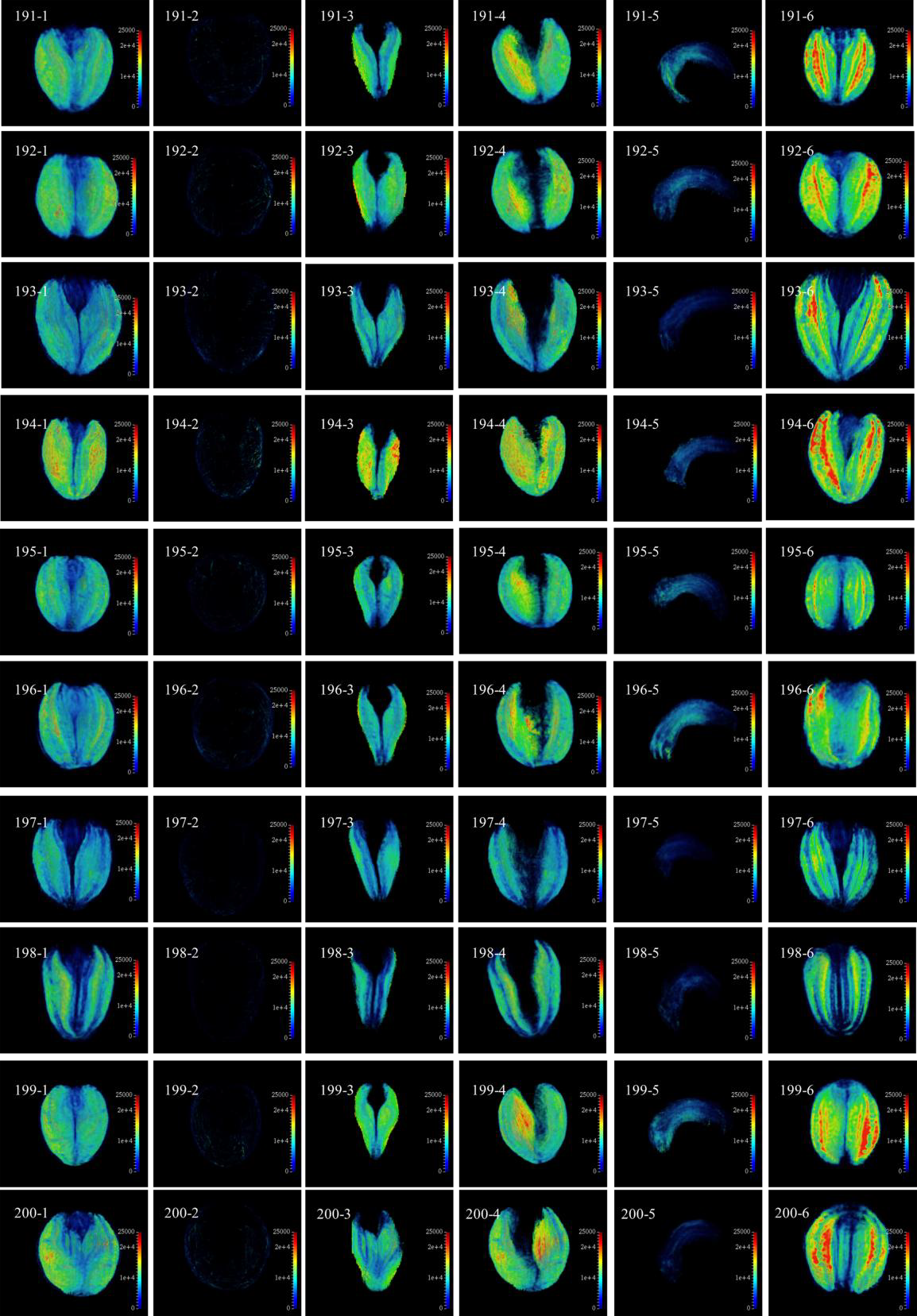


**Supplementary Fig 2.** Quantitative imaging of lipids in different tissues of seeds in KN DH population based on three dimensional reconstruction. 1, 2, 3, 4, 5 and 6 represent whole seed, seed coat, inner cotyledon, outer cotyledon, radicle and seed section, respectively.


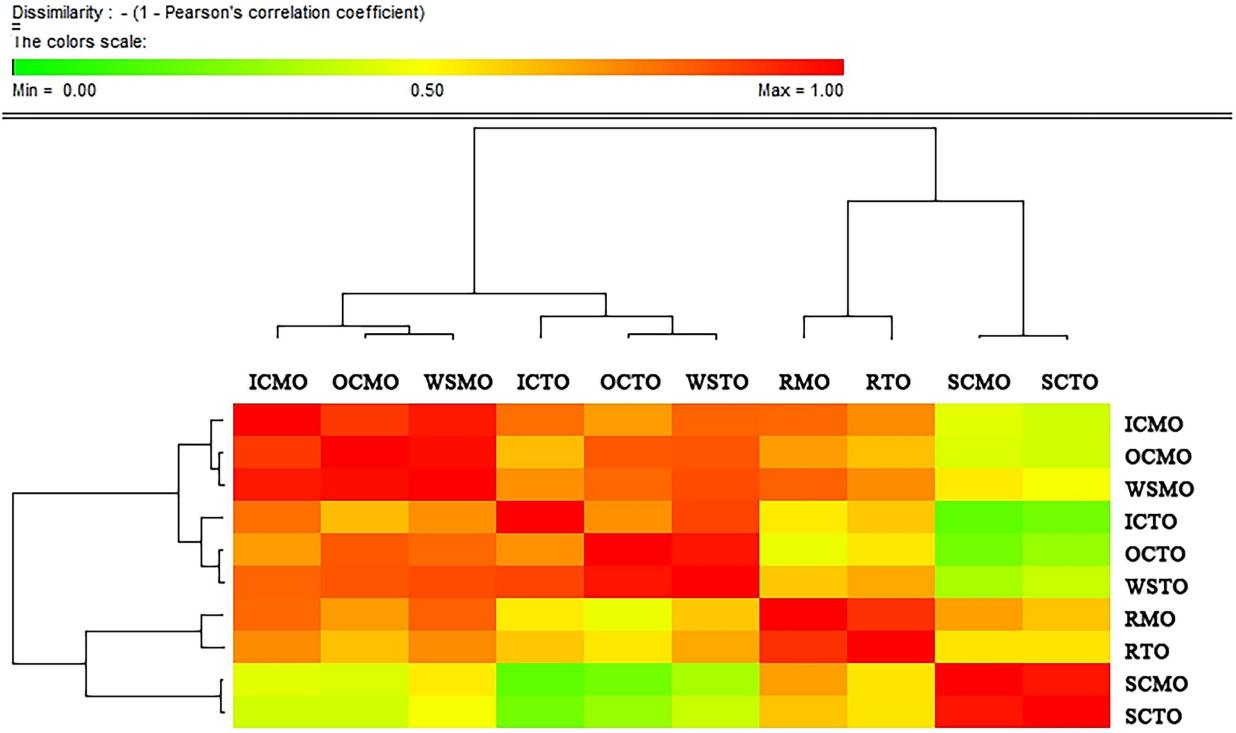


**Supplementary Figures 3.** Pearson correlation coefficients for trait pairs affecting oil content of rapeseed seeds in KN population.


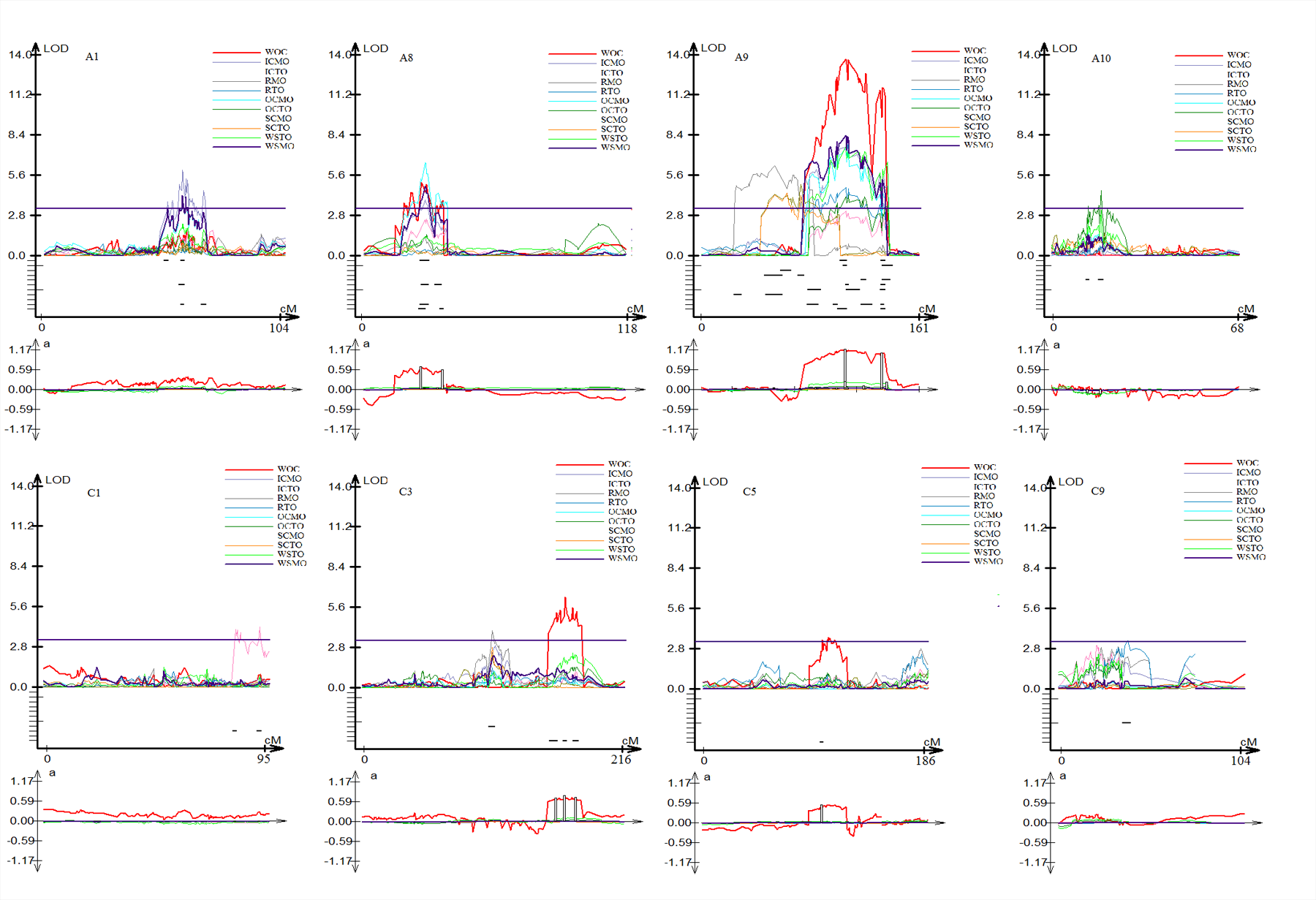


**Supplementary Figures 4.** Distribution of identified QTLs for oil content in different tissues of seeds on A1, A8, A9, A10, C1, C3 and C9 linkage group. WOC refer to identified QTLs for relative oil content detected by near infrared spectroscopy.

**Supplementary Figures 5.** The correlations among all 48 samples in different tissues at the two seed sampling stage of Ken-C8 and N53-2.


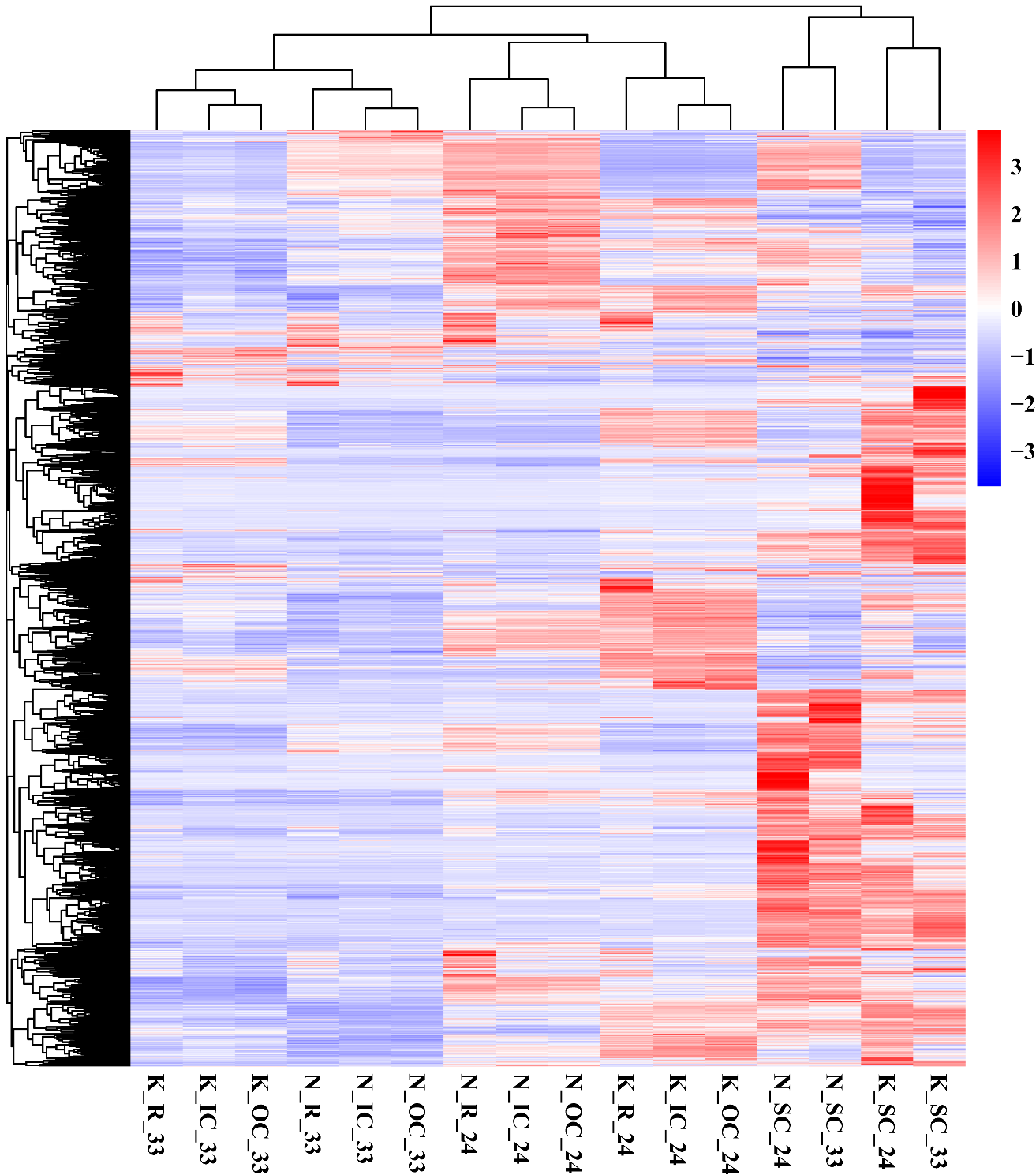


**Supplementary Figures 6.** The cluster analysis for 16 samples based on gene expression.

.


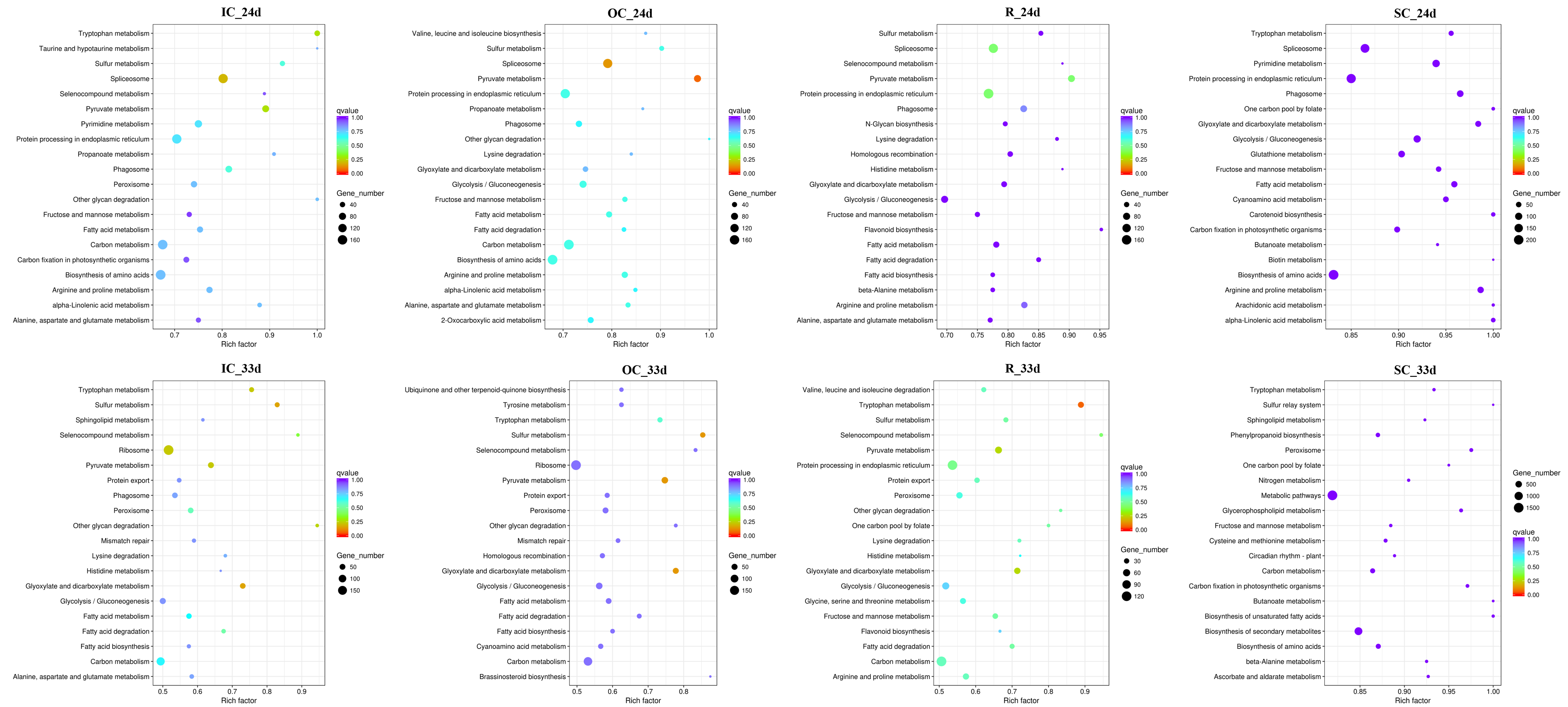


**Supplementary Figures 7.** KEGG enrichment of the tissues-specific DEGs (Ken-C8 vs N53-2) in the four tissues at 24 and 33 DAP.


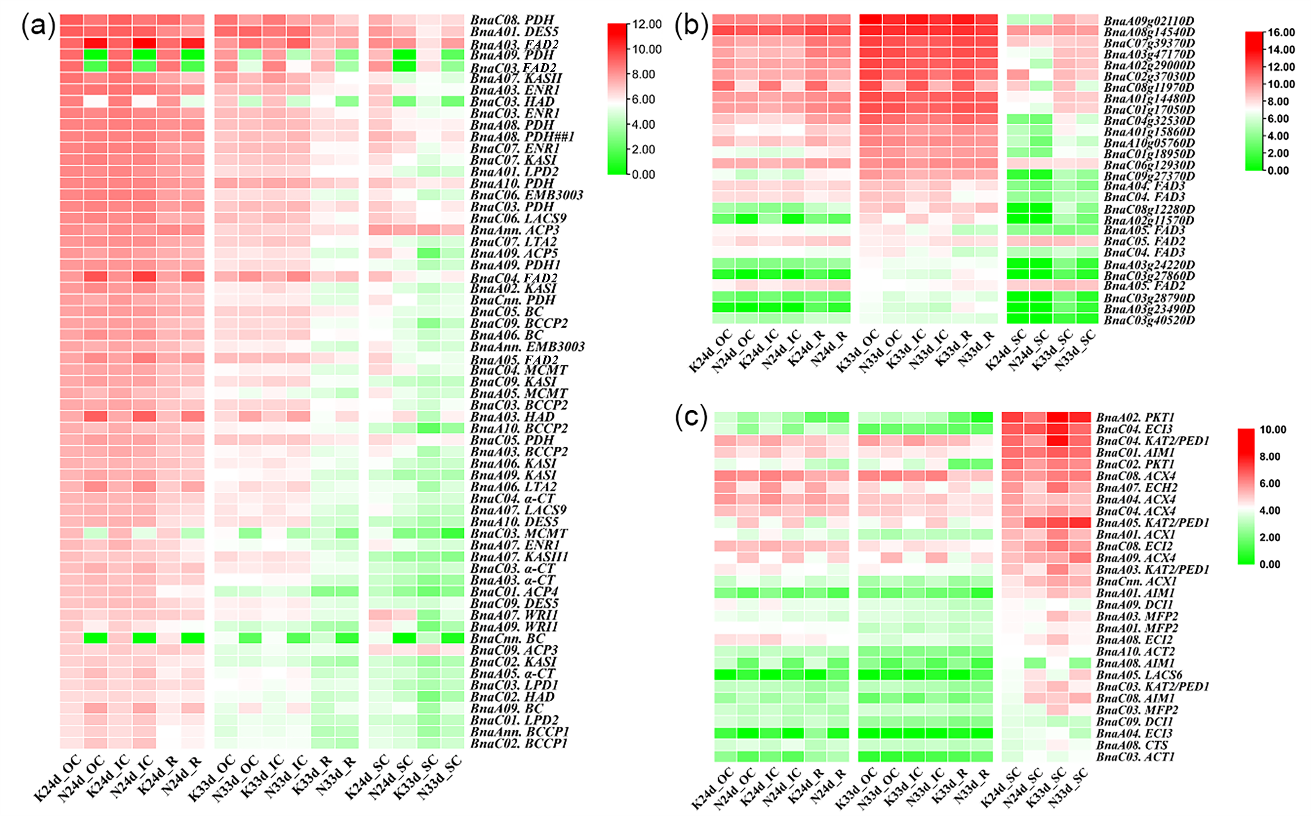


**Supplementary Figures 8.** The expression characteristics of genes involved in fatty acid synthesis (a), TAG synthesis (b) and β-oxidation (c) in four tissues of Ken-C8 and N53-2 at 24 and 33 DAF.
